# Differential Binding of Mitochondrial Transcripts by MRB8170 and MRB4160 Regulates Distinct Editing Fates of Mitochondrial mRNA in Trypanosomes

**DOI:** 10.1128/mBio.02288-16

**Published:** 2017-01-31

**Authors:** Sameer Dixit, Michaela Müller-McNicoll, Vojtěch David, Kathi Zarnack, Jernej Ule, Hassan Hashimi, Julius Lukeš

**Affiliations:** a Institute of Parasitology, Biology Center, Czech Academy of Sciences, Ceskě Budějovice, Czech Republic; b Faculty of Sciences, University of South Bohemia, Ceskě Budějovice, Czech Republic; c Institute for Cell Biology and Neuroscience, Goethe University, Frankfurt, Germany; d Buchmann Institute for Molecular Life Sciences, Goethe University, Frankfurt, Germany; e Department of Molecular Neuroscience, UCL Institute of Neurology, London, United Kingdom; f Canadian Institute for Advanced Research, Toronto, Ontario, Canada; University of Oxford

## Abstract

A dozen mRNAs are edited by multiple insertions and/or deletions of uridine residues in the mitochondrion of *Trypanosoma brucei*. Several protein complexes have been implicated in performing this type of RNA editing, including the mitochondrial RNA-binding complex 1 (MRB1). Two paralogous novel RNA-binding proteins, MRB8170 and MRB4160, are loosely associated with the core MRB1 complex. Their roles in RNA editing and effects on target mRNAs are so far not well understood. In this study, individual-nucleotide-resolution UV-cross-linking and affinity purification (iCLAP) revealed a preferential binding of both proteins to mitochondrial mRNAs, which was positively correlated with their extent of editing. Integrating additional *in vivo* and *in vitro* data, we propose that binding of MRB8170 and/or MRB4160 onto pre-mRNA marks it for the initiation of editing and that initial binding of both proteins may facilitate the recruitment of other components of the RNA editing/processing machinery to ensure efficient editing. Surprisingly, MRB8170 also binds never-edited mRNAs, suggesting that at least this paralog has an additional role outside RNA editing to shape the mitochondrial transcriptome.

## IMPORTANCE

*Trypanosoma brucei* mitochondrial mRNAs undergo maturation by RNA editing, a unique process involving decrypting open reading frames by the precise deletion and/or insertion of uridine (U) residues at specific positions on an mRNA. This process is catalyzed by multiprotein complexes, such as the RNA editing core complex, which provides the enzymatic activities needed for U insertion/deletion at a single editing site. Less well understood is how RNA editing occurs throughout an mRNA bearing multiple sites. To address this question, we mapped at single-nucleotide resolution the RNA interactions of two unique RNA-binding proteins (RBPs). These RBPs are part of the mitochondrial RNA-binding complex 1, hypothesized to mediate multiple rounds of RNA editing. Both RBPs were shown to mark mRNAs for the process in correlation with the number of editing sites on the transcript. Surprisingly, one also binds mRNAs that bypass RNA editing, indicating that it may have an additional role outside RNA editing.

## INTRODUCTION

*Trypanosoma brucei*, the causative agent of African sleeping sickness, is distinguished by a single reticulated mitochondrion containing an unusually large amount of mitochondrial DNA (mtDNA), termed kinetoplast DNA (kDNA). The kDNA comprises ~25 maxicircles and ~5,000 minicircles, mutually concatenated into a single network (1, 2). Maxicircles are homologs of classical mtDNA, containing two rRNAs and 18 protein-encoding genes, most of which constitute subunits of the mt respiratory complexes. Twelve out of 18 maxicircle mRNAs require numerous posttranscriptional insertions and/or deletions of uridine residues (U) to remove frameshifts and generate a correct open reading frame (3, 4). The kDNA minicircles are highly heterogeneous in sequence and carry small noncoding guide RNAs (gRNAs) (5). The binding of a gRNA to its cognate mRNA via Watson-Crick and G-U wobble base-pairing guides precise U insertions/deletions, eventually producing a fully edited mRNA (6).

The polycistronic maxicircle transcript is split into three differently processed transcript categories (7): (i) pan-edited mRNAs that undergo extensive editing mediated by several gRNAs in a 3′-to-5′ direction along the transcript (8), (ii) minimally edited mRNAs usually containing a single edited region, and (iii) never-edited mRNAs, which bypass editing and proceed directly to standard processing (9–11). However, little is known about how these individual transcripts, arising from a multicistronic precursor RNA, achieve distinct expression levels and how the abundance of these transcript categories is controlled in different life cycle stages of *T. brucei* (5, 10, 12).

Proteins are key components of the editing machinery, as they participate in all effector and regulatory steps in a highly coordinated manner (6, 10, 13). The RNA editing core complex (RECC), also called the editosome, is a large complex that contains the core enzymatic activities required for editing (14–16). Surprisingly, purified RECC is devoid of RNA and lacks processivity *in vitro* (17). Thus, additional proteins must cooperate with RECC to carry out multiple rounds of RNA editing *in vivo*. One such complex is the mtRNA-binding complex 1 (MRB1) (6). The MRB1 core complex is composed of six proteins: gRNA-associated proteins 1 and 2 (GAP1 and GAP2, respectively), plus MRB3010, MRB5390, MRB8620, and MRB11870 (18). This core is also referred to as the gRNA-binding complex (19) (for a guide to the different MRB1 protein nomenclatures in the field, see Table S1 in the supplemental material). The heterodimer of GAP1 and GAP2 was found to stabilize gRNAs (20, 21). Other vital MRB1 subunits are loosely associated with the core complex, including the accessory subunits MRB8170, MRB4160, and *T. brucei* RGG2 (TbRGG2) (6, 22). RNA interference (RNAi)-mediated depletion of most subunits leads to a profound decrease in pan-edited transcripts, while the effect on minimally edited mRNAs varies depending on the targeted subunits (18, 19, 23).

10.1128/mBio.02288-16.8 TABLE S1  MRB1 complex subunits. Listed are the proteins which were detected by various labs as comprising the MRB1 complex. Download TABLE S1, PDF file, 0.05 MB. Copyright © 2017 Dixit et al. 2017 Dixit et al. This content is distributed under the terms of the Creative Commons Attribution 4.0 International license.

MRB8170 and MRB4160 are unique RNA-binding proteins (RBPs), which were recently shown to bind RNA via a novel and hitherto-undefined RNA-binding domain (24). These proteins are highly similar paralogs that are conserved within the kinetoplastid flagellates but without orthologs outside this clade (24). Simultaneous depletion of MRB8170 and MRB4160 results in a decrease of edited forms of pan-edited and minimally edited transcripts and a slight increase in never-edited transcripts (24).

In this study, we used biochemical and genomics approaches to dissect the functions of MRB8170 and MRB4160 in processing different categories of maxicircle transcripts. We applied individual-nucleotide-resolution UV-cross-linking and affinity purification (iCLAP) (25, 26) to investigate interactions of both proteins with mtRNAs in the procyclic stage of *T. brucei*. This quantitative binding assay revealed a high preference of both proteins for maxicircle transcripts. Moreover, binding of both proteins influenced the steady-state abundance of mt mRNAs, as demonstrated by the double knockdown (dKD) of MRB8170 and MRB4160. Rapid tandem affinity purification (TAP) confirmed interaction of both proteins with the core and accessory MRB1 subunits GAP1 and TbRGG2, respectively (22, 27), and detected interactions with mtRNA-binding protein 1 (MRP1), Nudix hydrolase (or MERS1), and TbRGG1, which belong to different RNA processing complexes (10). Furthermore, the dKD of MRB8170 and MRB4160 was also shown to affect the mRNA-binding efficiency of these proteins. By integrating iCLAP data with *in vivo* and *in vitro* data, we propose the working dynamics of the MRB1 complex in facilitating RNA editing and also reveal a potential, unexpected role in the expression of never-edited transcripts.

## RESULTS

### MRB8170 and MRB4160 preferentially bind mitochondrial mRNAs.

**FIG 1:**
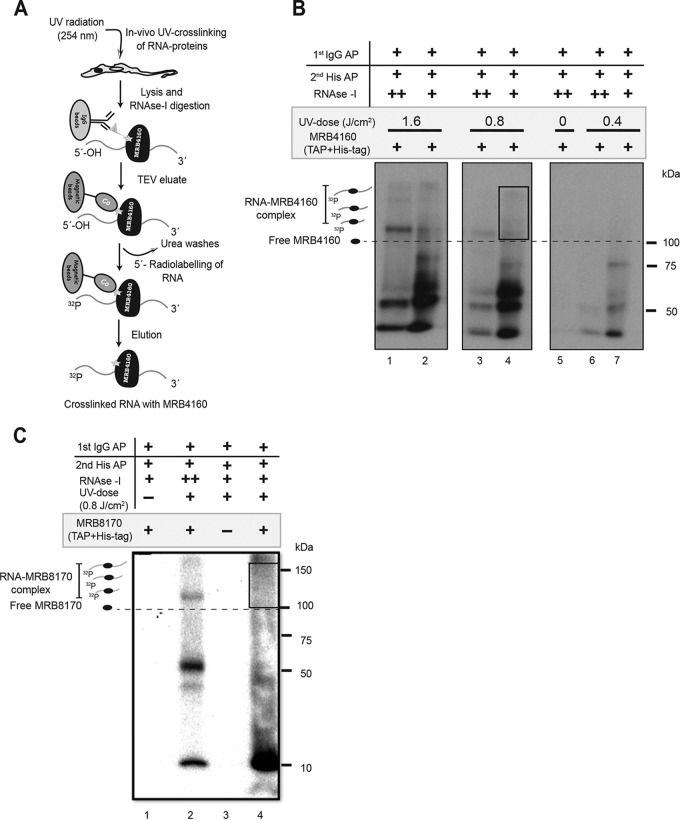
MRB4160 and MRB8170 iCLAP. (A) Schematic depiction of the iCLAP workflow to purify UV-cross-linked RNA-MRB4160-mTAP complex using two-step affinity purification. (B) Copurification of UV-cross-linked RNA-MRB4160-mTAP complex. Autoradiography of the ^32^P-labeled complexes after two-step affinity purification (AP). Three UV irradiant fluences were used: 1.6 J/cm^2^ (lanes 1 and 2), 0.8 J/cm^2^ (lanes 3 and 4), and 0.4 J/cm^2^ (lanes 6 and 7) to *in vivo* cross-link RNA with proteins, while non-UV-cross-linked cells (lane 5) were used as a control. The high (++)- and low (+)-RNase I treatments were applied to confirm the shift in the cross-linked RNA-MRB4160-mTAP complex under these conditions. The box marks the part that was cut out and used for RNA isolation. Two independent replicates were performed for preparation of the iCLAP library. (C) Copurification of UV-cross-linked RNA-MRB8170-mTAP complex. After two-step affinity purification, the ^32^P-labeled complexes were monitored by autoradiography. The optimal 0.8-J/cm^2^ UV radiant fluence was used to *in vivo* cross-link RNA to proteins. Non-UV-cross-linked mTAP-tagged MRB8170 (lane 1) and UV-cross-linked parental cell line (lane 3) yielded no signal. The high-RNase I treatment of UV-cross-linked MRB8170 (lane 2) showed a band at ~100-kDa size. The low-RNase treatment (boxed region in lane 4) was used to prepare the MRB8170 iCLAP libraries. Two independent replicates were performed for preparation of the iCLAP library.

We used the iCLAP protocol with the aim of identifying the direct RNA targets of the two accessory MRB1 subunits MRB8170 and MRB4160 in the mitochondrion of *T. brucei* (Fig. 1A). MRB4160 and MRB8170 were tagged with modified TAP tag (mTAP), bearing the His_6_ epitope, and stably expressed in *T. brucei* procyclic cells. In order to cross-link *in vivo* the tagged proteins to RNA, three UV irradiation doses (1.6, 0.8, and 0.4 J/cm^2^) were tested. Phosphorimaging of the cross-linked RNA revealed that UV cross-linking with a radiant energy ranging from 0.8 to 1.6 J/cm^2^ was more efficient than 0.4 J/cm^2^ (Fig. 1B; see also Fig. S1A in the supplemental material). Thus, a UV dose of 0.8 J/cm^2^ was applied for preparation of the MRB4160 and MRB8170 iCLAP libraries (Fig. 1C and S1B and C). No RNA-protein complexes were detected in the two controls, the non-UV-cross-linked trypanosomes with MRB4160-mTAP and the UV-cross-linked parental cells (Fig. 1B and C).

10.1128/mBio.02288-16.1 FIG S1  MRB8170 and MRB4160 iCLAP characterization. (A) Specificity and efficiency of affinity-purified, UV-cross-linked TAP-tagged MRB4160. The copurified UV-cross-linked RNA-MRB4160 complex after two-step affinity purification was confirmed by SDS-PAGE and Western blot analysis. The high-RNase I-treated samples from four different UV doses (1.6, 0.8, 0.4, and 0 J/cm^2^) were separately resolved using SDS-PAGE and transferred onto a nitrocellulose membrane, which was probed using anti-His antibody, recognizing an epitope on the modified TAP tag. MRB4160 was detected at its ~100-kDa size (lanes 1 to 4). **, partially degraded MRB4160 (modified TAP tag) protein. (B) Specificity and efficiency of affinity-purified, UV-cross-linked TAP-tagged MRB8170. The copurified low-RNase I-treated 0.8-J/cm^2^ UV-cross-linked MRB8170 with modified TAP tag was followed throughout two steps of affinity purification, fractions of which are indicated at the top. The membrane was probed as in panel A. MRB8170 migrated at ~100 kDa (lanes 1 to 7). **, partially degraded MRB8170 (modified TAP tag) protein. (C) Specificity and efficiency of affinity-purified, non-UV-cross-linked TAP-tagged MRB8170. The non-UV-cross-linked TAP-tagged MRB8170 and control parental cell lines were processed and assayed as in panels A and B. MRB8170 migrated at the expected ~100-kDa size (lanes 1 and 6), while there was no signal in the control (lane 3). Labeling as in panel B. (D) TBE-6% urea gel of the amplified iCLAP sequencing libraries from TAP-tagged MRB8170 and MRB4160, plus parental cell lines. The cDNA was size fractionated and amplified separately to reduce any size biases during PCR. Three sizes were cut out and PCR amplified, indicated as high (H) (150 to 300 nucleotides; lanes 1, 4, and 8), medium (M) (80 to 150 nucleotides; lanes 2, 5, and 9), and low (L) (60 to 80 nucleotides; lanes 3, 6, and 10). The H- and M-nucleotide-long libraries were submitted for next-generation sequencing. RT –ve, reverse transcriptase control (lane 7); MRB4160, iCLAP libraries from UV-cross-linked modified TAP-tagged MRB4160 (lanes 5, 6, and 7); MRB8170, iCLAP libraries from UV-cross-linked modified TAP-tagged MRB8170 (lanes 1, 2, and 3); 29-13 negative control (lanes 4, 5, and 6), iCLAP libraries from UV-cross-linked parental 29-13 strain (mock affinity purification). After the removal of adaptor sequence, the resulting iCLAP tags were ~30 to 50 bp in size. Download FIG S1, PDF file, 1.3 MB. Copyright © 2017 Dixit et al. 2017 Dixit et al. This content is distributed under the terms of the Creative Commons Attribution 4.0 International license.

Cross-linked and affinity-purified RNA from two independent iCLAP replicates with MRB8170-mTAP, MRB4160-mTAP, and the control (UV-cross-linked parental cells) was RNase I digested into 60- to 120-nucleotide (nt)-long fragments, reverse transcribed, and subjected to next-generation sequencing (Fig. S1D). The sequencing reads, henceforth referred to as iCLAP tags, were aligned against the preedited and fully edited versions of the kDNA maxicircle transcripts using Bowtie2 alignment software (28). The two replicates combined from MRB8170 and MRB4160 data sets yielded a total of 191,683 and 100,313 uniquely aligned iCLAP tags, respectively. The control library obtained from the UV-cross-linked parental cells contained only a negligible 483 unique iCLAP tags. This very low number of control iCLAP tags confirmed the high stringency of the applied iCLAP protocol.

### Promiscuous binding of MRB8170 to all classes of mitochondrial mRNAs contrasts with restricted binding of MRB4160.

**FIG 2:**
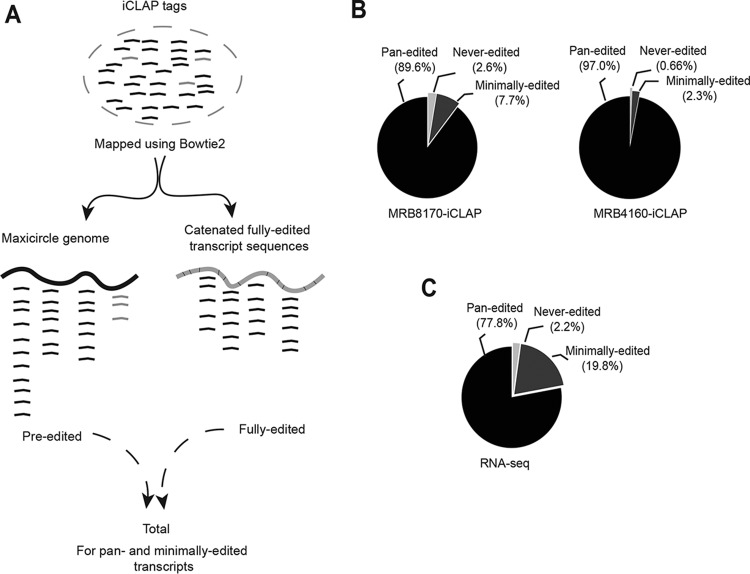
Distribution of MRB8170 and MRB4160 iCLAP tags on maxicircle transcripts. (A) Schema of the strategy to map iCLAP tags onto mitochondrial mRNAs. iCLAP tags were separately mapped to maxicircle genome and catenated sequences of fully edited transcripts (9 pan-edited mRNAs plus 3 minimally edited mRNAs). The tags uniquely mapped to maxicircle genome were named preedited, while iCLAP tags mapped to catenated sequences were categorized as fully edited (see text for explanation). For transcripts undergoing editing, preedited and fully edited mapped iCLAP tags (black) were combined for analysis. The iCLAP tags (gray) mapped to never-edited transcripts were limited to the preedited region. (B) Pie chart of uniquely mapped iCLAP tags on the maxicircle mRNAs. Percentages of MRB8170 and MRB4160 iCLAP tags uniquely mapped to three different classes of maxicircle mRNAs. Black, pan-edited mRNAs (preedited and fully edited iCLAP tags mapped to pan-edited region); dark gray, minimally edited mRNAs (preedited and fully edited iCLAP tags mapped to minimally edited region); light gray, never-edited mRNAs. Two independent iCLAP replicates each for MRB8170 and MRB4160 were combined for the analysis. (C) Percentage of RNA-seq tags uniquely mapped to the same classes of maxicircle mRNAs. The pie chart is shaded as in panel B.

To analyze the binding of MRB8170 and MRB4160 on maxicircle-derived transcripts, we divided the iCLAP tags into two categories according to their generation from preedited and fully edited transcripts (Fig. 2A). Since preedited iCLAP tags had been mapped directly to the maxicircle genome, they include all 18 maxicircle-derived pre-mRNAs (pan-edited, minimally edited, and never-edited mRNAs) before undergoing editing. In contrast, fully edited iCLAP tags had been mapped to 12 fully edited maxicircle mRNAs (pan-edited and minimally edited) in which all U insertions/deletions had been completed.

To dissect RNA interactions of RBPs that are part of large stable protein complexes, such as MRB1, it is necessary to use extended RNase I digests to generate small RNA fragments. Our protocol produced iCLAP tags ~30 to 50 nt long after the removal of the adaptor sequences. However, a drawback of the short read length is that iCLAP tags mapping to fully edited sequences can also be derived from partially edited mRNAs still undergoing the process. Vice versa, iCLAP tags mapping to preedited sequences can originate from RNAs not yet edited, or from already partially edited transcripts. Thus, in both cases, it is impossible to quantitate the amount of reads originating from partially edited mRNAs, which creates a bias in the numbers of preedited and fully edited iCLAP tags (Fig. S2A). Approximately 95.3% of MRB4160 iCLAP tags aligned to preedited mRNAs, while 4.6% aligned to fully edited mRNAs (Fig. S2A). Similarly, 90.7% and 9.2% of MRB8170 iCLAP tags mapped to preedited and fully edited mRNAs, respectively (Fig. S2A).

10.1128/mBio.02288-16.2 FIG S2  iCLAP and RNA-seq binding to maxicircle. (A) Pie chart of uniquely mapped iCLAP tags (~30 to 50 nt long) and RNA-seq reads mapped to the maxicircle genome. Percentage of MRB8170 iCLAP tags, MRB4160 iCLAP tags, and RNA-seq reads mapped to preedited and fully edited maxicircle transcripts. Preedited, black; fully edited, gray. Never-edited transcripts were excluded for the analyses. Our protocol successfully obtained ~30- to 50-nt-long iCLAP tags, after the removal of the adaptor sequences. A drawback of the short read length of iCLAP tags is the resulting inability to quantitate the amount of reads originating from partially edited mRNAs; therefore, in both cases, it is impossible to quantitate the amount of reads originating from partially edited mRNAs, which creates a bias in the number of preedited and fully edited transcripts in the iCLAP pie chart. (B) Percentage of RNA-seq reads uniquely mapped to the three classes of maxicircle mRNAs. Three bar plots showing the percentage of uniquely mapped RNA-seq reads to pan-edited transcripts in the preedited and fully edited regions, plus the total of the two (preedited and fully edited), respectively, for each of the mtRNAs indicated on the *x* axis. (C) Normalized bar plot with respect to gene length of never-edited transcripts. Three bar plots show the normalized data (using DEX-seq) with respect to gene length of MRB8170 iCLAP tags, MRB4160 iCLAP tags, and RNA-seq reads mapped to never-edited transcripts of the mt mRNA indicated on the *x* axis. Download FIG S2, PDF file, 1 MB. Copyright © 2017 Dixit et al. 2017 Dixit et al. This content is distributed under the terms of the Creative Commons Attribution 4.0 International license.

Next, we used our quantitative iCLAP data to establish the proportion of binding relative to the extent of RNA editing. For this, maxicircle mRNAs were divided into pan-edited (*COX3*, *ND7*, *ND8*, *A6*, *CR3*, *RPS12*, *ND9*, *ND3*, and *CR4*), minimally edited (*COX2*, *MURF2*, and *CYB*) and never-edited (*ND1*, *COX1*, *ND4*, *ND5*, *MURF5*, and *MURF1*) transcript categories. For those transcripts undergoing editing, preedited and fully edited iCLAP tags were combined. The distribution of MRB8170 and MRB4160 iCLAP tags on mtRNAs was compared to their expression level determined by publicly available *T. brucei* RNA sequencing (RNA-seq) data, which also include transcripts originating from the organelle (29).

**FIG 3:**
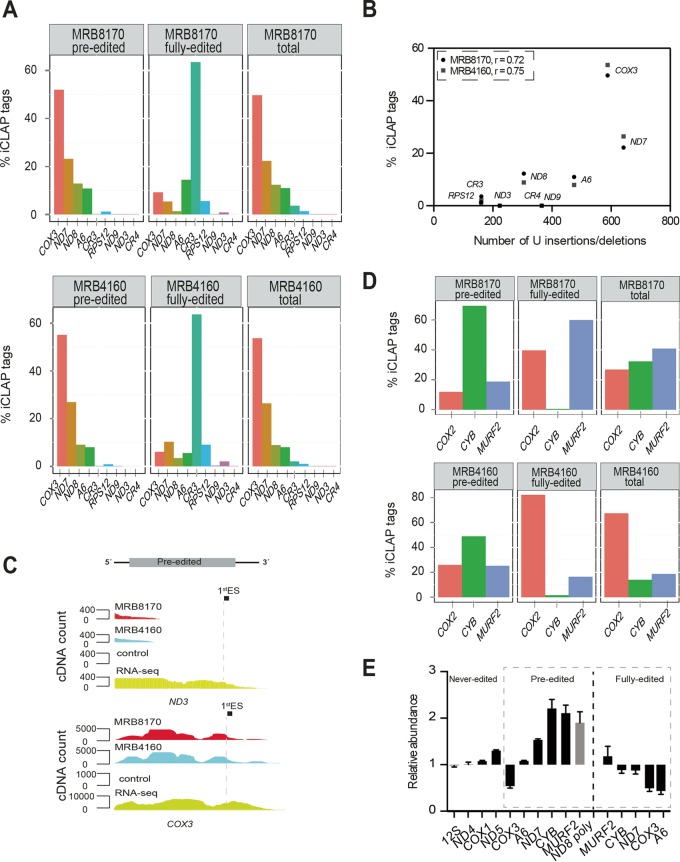
MRB8170 and MRB4160 binding to transcripts undergoing editing. (A) Preferential binding of MRB8170 and MRB4160 to pan-edited transcripts. Bar plots show the percent share of MRB8170 and MRB4160 iCLAP tags uniquely mapped to the preedited and fully edited regions of pan-edited transcripts and the total (preedited and fully edited), respectively, of pan-edited mRNAs indicated on the *x* axis. (B) Scatter plot depicting the correlation between total share of mapped iCLAP tags (*y* axis) and the number of U insertions or deletions, reflecting the extent of editing of individual transcripts (*x* axis). Each point represents a pan-edited transcript (*RPS12*, *CR3*, *ND3*, *CR4*, *ND8*, *ND9*, *A6*, *COX3*, and *ND7*) as indicated. Pearson’s correlation coefficients (*r*) are shown for both MRB8170 and MRB4160. Black circles, MRB8170; gray squares, MRB4160. (C) Genomic browser view displays preferential binding of MRB8170 and MRB4160 to *ND3* and *COX3* transcripts. The unique cDNA count is depicted on the *y* axis, and the mapped tag position along a given transcript is on the *x* axis. MRB8170 iCLAP tags are in red, MRB4160 iCLAP tags are in blue, control iCLAP tags are in black, and RNA-seq reads are in yellow. ES, editing site. (D) Binding of MRB8170 and MRB4160 to minimally edited transcripts. Labeling as in panel A. (E) Relative abundance of maxicircle mRNAs compared between MRB8170/MRB4160 and ATM1 knockdown cells by qPCR analysis. 18S rRNA was used as an internal reference. The following maxicircle mRNAs were analyzed in triplicate: rRNA (12S), never-edited mRNA (*ND4*, *COX1*, and *ND5*), pan-edited mRNA (*COX3*, *A6*, and *ND7*), minimally edited mRNA (*CYB* and *MURF2*), and *ND8* poly (polycistronic *ND8* transcript). The dashed line separates preedited and fully edited versions of the transcripts. All mRNAs are in black except *ND8* poly, which is in gray.

Interestingly, the proportion of MRB8170 iCLAP tags that map onto never-edited RNAs (2.6%) correlates with their occurrence in the RNA-seq data (2.2%) (Fig. 2B and C). In contrast, a surprisingly high fraction (~97%) of MRB4160 iCLAP tags mapped to pan-edited transcripts, while binding to never-edited transcripts was negligible (Fig. 2B). This result was confirmed by RNA immunoprecipitation (RIP)-quantitative real-time PCR (qPCR) (Fig. 3E). In summary, our data suggest that MRB8170 binds all classes of maxicircle mRNAs, while MRB4160 binding is restricted to pan-edited and minimally edited transcripts.

### MRB8170 and MRB4160 binding on pan-edited and minimally edited transcripts correlates with their editing status.

In order to understand the function(s) of MRB8170 and MRB4160 in editing, we quantified the binding of both proteins to nine individual pan-edited transcripts using iCLAP tags mapping to preedited and fully edited mRNAs (Fig. 3A). In agreement with being paralogs, the distributions of MRB8170 and MRB4160 iCLAP tags mapping onto pan-edited transcripts were very similar (Fig. 3A). For instance, both proteins massively bind to preedited *COX3* but have minimal binding to *ND9*, *ND3*, and *CR4*. Interestingly, the extent of binding correlates with the number of U insertions/deletions needed to be fully edited (Fig. 3B). Visual inspection of iCLAP tags in the genome browser showed that both proteins bind continuously along the entire preedited sequence of six out of nine pan-edited transcripts, including *A6*, *CR3*, *COX3*, *ND7*, *ND8*, and *RPS12* (Fig. 3C and S3 and S4). Since RNA editing proceeds in a stepwise manner in a 3′-to-5′ direction, the pronounced binding of MRB8170 and MRB4160 over the entire length of these preedited transcripts hinted at their role in flagging pan-edited RNAs for editing.

10.1128/mBio.02288-16.3 FIG S3  MRB8170 and MRB4160 binding to a subset of pan-edited transcripts (*ND3*, *ND9*, *CR4*, *COX3*, and *RPS12*). Genomic browser snapshot of iCLAP tags and RNA-seq reads mapped to individual pan-edited transcripts, for which the preedited and fully edited versions are shown separately. The unique cDNA count is depicted on the *y* axis, and the mapped tag position along a given transcript is shown on the *x* axis. MRB8170 iCLAP tags are in red, MRB4160 iCLAP tags are in blue, control iCLAP tags are in black, and RNA-seq reads are in yellow. *ND3*, *ND9*, and *CR4* lack significant iCLAP tags from both MRB8170 and MRB4160 to preedited sequence. The other 6 pan-edited mRNAs (*COX3*, *RPS12*, *CR3*, *ND8*, *ND7*, and *A6*) shared various orders of iCLAP tags from MRB8170 and MRB4160, positively correlating with their editing extent. Download FIG S3, PDF file, 1.5 MB. Copyright © 2017 Dixit et al. 2017 Dixit et al. This content is distributed under the terms of the Creative Commons Attribution 4.0 International license.

10.1128/mBio.02288-16.4 FIG S4  MRB8170 and MRB4160 binding to a subset of pan-edited transcripts (*CR3*, *ND8*, *ND7*, and *A6*). Labeling as in Fig. S3. Download FIG S4, PDF file, 1.6 MB. Copyright © 2017 Dixit et al. 2017 Dixit et al. This content is distributed under the terms of the Creative Commons Attribution 4.0 International license.

In contrast, both MRB8170 and MRB4160 showed strong accumulation toward the 5′ end of the preedited *ND3* and *CR4* mRNAs and minimal binding to *ND9* mRNA (Fig. 3C and S3), although they are well expressed, as judged from RNA-seq data (Fig. S2B). This observation suggests that these transcripts are not flagged for editing by MRB8170 and MRB4160. The observation that the preedited forms of some pan-edited transcripts are completely covered by MRB8170 and MRB4160, while others show binding only toward the 5′ end, could explain previous reports on different editing states in the procyclic stage of *T. brucei*. Indeed, the two paralogs bind the entire length of preedited *COX3*, *RPS12*, and *A6* mRNAs, which are all fully edited in the procyclic stage. Furthermore, their limited binding onto preedited *ND3* and *CR4* mRNAs correlates with their not being edited in this stage (30–32).

Next, we dissected the binding of MRB8170 and MRB4160 to the minimally edited *COX2*, *CYB*, and *MURF2* transcripts, which have 4, 39, and 26 U insertions, respectively, plus four U deletions in the case of *MURF2*. Binding of MRB8170 and MRB4160 to fully edited *CYB* was extremely low (Fig. 3D). As this transcript also exhibits a low steady-state level in the mt transcriptome (Fig. S2B), low binding likely reflects the paucity of fully edited *CYB* in the procyclic stage. In contrast, both proteins bind over the entire length of preedited *COX2*, *CYB*, and *MURF2* transcripts (Fig. 3D and S5), suggesting that they mark all three minimally edited transcripts for editing, similarly to pan-edited mRNAs.

10.1128/mBio.02288-16.5 FIG S5  MRB8170 and MRB4160 binding to minimally edited transcripts. Genomic browser snapshot of iCLAP tags and RNA-seq reads mapped to *COX2*, *MURF2*, and *CYB* transcripts. Labeling as in Fig. S3. The genome browser snapshots depicted the binding of proteins over the entire preedited *COX2*, *MURF2*, and *CYB* transcripts. Download FIG S5, PDF file, 1.1 MB. Copyright © 2017 Dixit et al. 2017 Dixit et al. This content is distributed under the terms of the Creative Commons Attribution 4.0 International license.

To further validate the impact of both proteins on editing of pan-edited and minimally edited transcripts, we assayed the relative abundance of maxicircle transcripts by quantitative real-time PCR (qPCR) in MRB8170/MRB4160 dKD cells. Control cells were depleted of ATM1 mRNA, encoding an inner membrane transporter that does not affect mt gene expression (33). Indeed, qPCR analysis showed that preedited forms of pan-edited and minimally edited mRNAs accumulated upon MRB8170/MRB4160 depletion, but not in control cells, while the relative abundance of fully edited transcripts was considerably reduced (Fig. 3E).

Taken together, iCLAP and knockdown data support a role for MRB8170 and MRB4160 in flagging mRNAs for editing, as their absence reduces the abundance of edited transcripts in the procyclic stage.

### MRB8170 binds to a subset of less-abundant never-edited transcripts.

**FIG 4:**
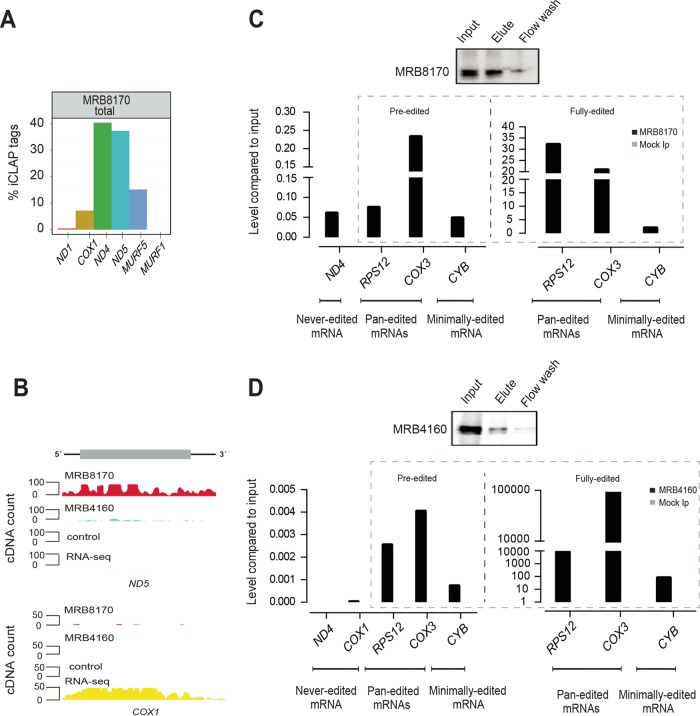
MRB8170 and MRB4160 binding to never-edited transcripts and iCLAP validation by RNA immunoprecipitation-quantitative PCR. (A) Preferential binding of MRB8170 to never-edited transcripts. The bar plot shows the percent share of MRB8170 iCLAP tags uniquely mapped to never-edited transcripts, as indicated on the *x* axis. (B) Genomic browser view displays preferential binding of MRB8170 and MRB4160 to *ND5* and *COX1* transcripts. The unique cDNA count is depicted on the *y* axis, and the mapped tag position along a given transcript is on the *x* axis. MRB8170 iCLAP tags are in red, MRB4160 iCLAP tags are in blue, control iCLAP tags are in black, and RNA-seq reads are in yellow. (C) MRB8170-associated maxicircle transcripts determined by RIP-qPCR. The top panel confirms MRB8170 purification using IgG beads by Western blotting. Bar plots below show the relative amount of never-edited (*ND4*), pan-edited (*RPS12* and *COX3*), and minimally edited (*CYB*) mRNAs pulled down with mTAP-tagged MRB8170 (black) and mock immunoprecipitation (Mock Ip, parental cell line, in gray). Data are presented relative to the input sample (RNA recovered and reverse transcribed using 10% of lysate). One representative set of measurements is shown. (D) MRB4160-associated maxicircle transcripts determined by RIP-qPCR. Labeling as in panel C.

We next investigated binding of MRB8170 to six never-edited transcripts. *ND4*, *ND5*, and *MURF5* mRNAs were represented in more than 90% of the iCLAP tags mapping to never-edited transcripts, while the remainder were derived from *ND1*, *COX1*, and *MURF1* (Fig. 4A). Normalization of the iCLAP tag number to gene length resulted in similar proportions of iCLAP tags (Fig. S2C).

Such biased binding of MRB8170 to a subset of never-edited transcripts was unexpected and prompted us to look into their steady-state relative abundances. Interestingly, *ND1* and *COX1* are the most abundant never-edited transcripts in procyclic trypanosomes (Fig. 4B and S6) (30–32). Hence, there is a notable discrepancy between the very low number of iCLAP tags and the high expression of these two genes. For *MURF1* on the other hand, the insignificant number of mapped iCLAP tags corresponds to its low abundance, rendering its detection difficult by both iCLAP and RNA-seq methods. In contrast, the enrichment of *ND4*, *ND5*, and *MURF5* bound to MRB8170 did not correspond to their relatively low steady-state levels as determined by RNA-seq (Fig. 4B, S2B, and S6). This result suggests that MRB8170 serves an additional role outside RNA editing by negatively regulating the expression of this subset of never-edited transcripts. This notion is supported by the accumulation of never-edited transcripts in MRB8170/MRB4160 dKD cells (Fig. 3E).

10.1128/mBio.02288-16.6 FIG S6  MRB8170 and MRB4160 preferential binding to never-edited transcripts. Genomic browser snapshot of iCLAP tags and RNA-seq reads mapped to *ND4*, *MURF5*, *ND1*, and *MURF1* transcripts. Labeling as in Fig. S3. The other two never-edited transcripts (*ND4* and *COX1*) are part of the main figure (Fig. 4B). *ND1* and *COX1* lack significant iCLAP tags, whereas both show significant RNA-seq reads. Download FIG S6, PDF file, 1 MB. Copyright © 2017 Dixit et al. 2017 Dixit et al. This content is distributed under the terms of the Creative Commons Attribution 4.0 International license.

To validate the iCLAP data, we performed RNA immunoprecipitation (RIP) in cell lines expressing tagged MRB8170-mTAP or MRB4160-mTAP and a parental cell line lacking the mTAP-tag (mock IP). Immunoprecipitated RNA was reverse transcribed, and qPCR was performed using primers recognizing preedited and fully edited versions of pan-edited *RPS12* and *COX3*, minimally edited *CYB*, and never-edited *ND4* and *COX1* transcripts. These data confirmed that MRB8170 binds to all three classes of maxicircle mRNAs similarly enriched for never-edited (*ND4*) and pan-edited (preedited *RPS12*) mRNAs. As seen before in the iCLAP data, MRB4160 failed to bind to never-edited transcripts (Fig. 4C and D).

### MRB8170 and MRB4160 interact with non-MRB1 proteins.

**FIG 5:**
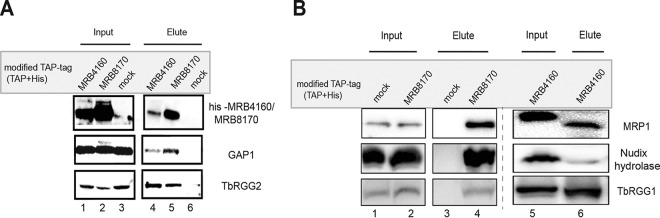
MRB8170- and MRB4160-associated proteins. (A) Rapid affinity purification of MRB8170- and MRB4160-associated proteins belonging to the MRB1 complex. Western blot analysis of proteins indicated on the right in total extracts (input; lanes 1 to 3) and eluates (lanes 4 and 5). The control includes a mock purification of the parental cell line (lanes 3 and 6). (B) Confirmation of rapid affinity purification of MRB8170- and MRB4160-associated proteins belonging to different RNA processing complexes. Western blot analysis of indicated proteins in total extracts (input, lanes 1, 2, and 5) and eluates (lanes 3, 4, and 6). The control includes a mock purification of the parental cell line (lanes 1 and 3).

After identification of MRB8170 and MRB4160 RNA-binding sites and finding that the former binds to never-edited transcripts, we wondered about their interactions with non-MRB1 proteins involved in RNA editing or other RNA processing steps. For this purpose, we performed rapid tandem affinity purification using IgG-coated magnetic Dynabeads (34). RNase I-digested supernatants from *T. brucei* containing mTAP-tagged MRB8170 or MRB4160, as well as the parental control cell line, were mixed with the beads. In order to validate this new protocol for its pulldown efficiency, the eluates were first probed with antibodies against GAP1 and TbRGG2 from MRB1, which are known to stably interact with MRB8170 and MRB4160 (6). Indeed, both GAP1 and TbRGG2 were detected in MRB8170 and MRB4160 pulldowns, while their absence in the control demonstrated the high stringency of this approach (Fig. 5A). Eluates were then probed with a panel of specific antibodies revealing additional interactions of both proteins with MRP1 from the MRP1/MRP2 complex and with Nudix hydrolase and TbRGG1 (Fig. 5B). All proteins are part of complexes with known roles in stabilizing RNA (13, 19, 35).

### Depletion of MRB8170 and MRB4160 affects RNA-binding activity of interacting proteins.

**FIG 6:**
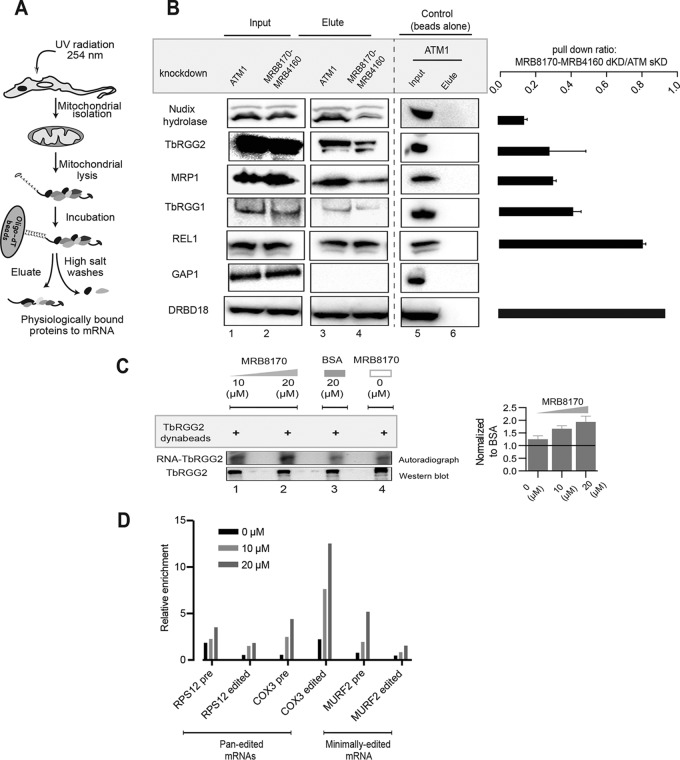
mRNA-binding efficiency of associated proteins following MRB8170/MRB4160 double knockdown. (A) Workflow of *in vivo* UV cross-linking and oligo(dT) magnetic bead pulldown of mitochondrial proteins associated with maxicircle mRNAs. (B) Western blot analysis of total extracts (lanes 1 and 2) and oligo(dT) eluates (lanes 3 and 4) from ATM1 and MRB8170/MRB4160 RNAi knockdown cells displaying levels of proteins indicated on the left. The control is beads not conjugated to oligo(dT) (lanes 5 and 6). Bar plots on the right show the ratio of lane 4 to lane 3 (MRB8170/MRB4160 versus ATM1 knockdown cell pulldown efficiency) signals for each protein. Error bars represent standard deviations (*n* = 2 to 3 for all proteins and *n* = 1 for DRBD18). sKD, single knockdown. (C) *In vitro* CLIP assay in MRB8170/MRB4160 double-knockdown cells. The ^32^P-labeled RNA from the immunoprecipitated TbRGG2-RNA complex was visualized by autoradiography (autoradiograph; lanes 1 to 4). Below is a Western blot showing the eluted TbRGG2 (Western blot; lanes 1 to 4). A consistent amount of supernatant from MRB8170/MRB4160-depleted cells was supplemented with recombinant GST-MRB8170, indicated above in micromolar concentrations (lanes 1, 2, and 4) and BSA (20 µM; lane 3). The bar plot was calculated relative to the BSA (*n* = 2); error bars, standard deviations. (D) *In vitro* CLIP assay and subsequent qPCR analyses in MRB8170/MRB4160 double-knockdown cells labeled as in panel C. Bar plots show the relative amounts of *RPS12*, *COX3*, and *MURF2* in both preedited and fully edited forms as obtained from TbRGG2 pulldown in MRB8170/MRB4160-depleted cells supplemented with recombinant GST-MRB8170, indicated above in micromolar concentrations (lanes 1, 2, and 4) and BSA (20 µM; lane 3). The bar plot was calculated relative to BSA. A representative set of measurements is shown.

We modified the protocol for UV cross-linking and subsequent pulldown of RBPs using oligo(dT) magnetic beads (36) in *T. brucei*, using the same UV dose as applied in iCLAP (Fig. 6A). The modified protocol to capture the RBPs was applied to the procyclic stage, in which we depleted either MRB8170/MRB4160 by dKD or ATM1 as a negative control (33). Oligo(dT)-captured RBPs were resolved by SDS-PAGE and transferred onto a nitrocellulose membrane. Subsequently, the mt mRNA interactome was probed with antibodies specific for Nudix hydrolase, TbRGG2, MRP1, TbRGG1, the RECC subunit RNA editing ligase 1 (REL1), and GAP1 (Fig. 6B and S7A). REL1 was the only examined protein without a significant reduction in the pulldown ratio between MRB8170/MRB4160 and ATM1 depletion (Fig. 6B). Nudix hydrolase and TbRGG2 exhibited the highest decrease in poly(A)^+^ RNA binding upon MRB8170/MRB4160 depletion. Captured MRP1 and TbRGG1 proteins were reduced to a lesser degree but still by more than 50% (Fig. 6B). The absence of GAP1 in our cross-linked mt mRNA interactome pulldown suggests that its RNA binding *in vivo* is strictly limited to gRNAs. As a control, we assessed the poly(A)^+^ RNA binding of the cytoplasmic mRNA-binding protein DRBD18 (29), which as expected was not affected by the depletion of MRB8170 and MRB4160.

10.1128/mBio.02288-16.7 FIG S7  MRB8170- and MRB4160-associated interactome. (A) Western blot analysis of total extracts (lanes 1 and 2) and oligo(dT) eluates (lanes 3 and 4) from MRB8170/MRB4160 and ATM1 RNAi-induced knockdown cells. GAP1 and MRP1 were detected in parallel using the same polyvinylidene difluoride membrane to serve as a loading control for the oligo(dT) experiment. (B) *In vitro* CLIP assay from Fig. 5. (C) The ^32^P-labeled RNA from immunoprecipitated TbRGG2-RNA complex was visualized by autoradiography (autoradiograph; lanes 1 to 4). The untrimmed autoradiography film is shown. Download FIG S7, PDF file, 1.6 MB. Copyright © 2017 Dixit et al. 2017 Dixit et al. This content is distributed under the terms of the Creative Commons Attribution 4.0 International license.

The decrease in the mt mRNA-binding efficiency of TbRGG2 caused by the depletion of MRB8170 and MRB4160 was further validated using an *in vitro* cross-linking immunoprecipitation (CLIP) assay, using extracts from MRB8170/MRB4160-depleted cells lysed under mild conditions. The lysate was divided into four equal aliquots and subsequently supplemented with recombinant glutathione *S*-transferase (GST)-tagged MRB8170 (10 and 20 µM), bovine serum albumin (BSA) (20 µM), or buffer alone. The supplemented supernatant was incubated and subsequently *in vitro* UV cross-linked. TbRGG2 antibody-coated magnetic beads were used to pull down the protein-RNA adducts, followed by 5′ radioactive labeling of the bound nucleic acid. Upon resolution in an SDS-PAGE gel, the immunoprecipitated RNA-TbRGG2 complex was transferred onto a nitrocellulose membrane. The resulting autoradiogram depicted a direct relationship between the supplemented recombinant GST-MRB8170 in the supernatant and the amount of RNA bound to TbRGG2 (Fig. 6C and S7B). The addition of BSA into the supernatant as a control also caused a slight decrease in the intensity of the autoradiogram signal, which may be a consequence of nonspecific binding of BSA onto the beads. The notion that recombinant GST-MRB8170 enhances TbRGG2 RNA binding *in vitro* was further substantiated by using RIP-qPCR to show TbRGG2 binding to several minimally and pan-edited mRNAs (Fig. 6D).

Taken together, the *in vivo* and *in vitro* data confirmed the role of MRB8170 and MRB4160 in mediating efficient binding of Nudix hydrolase, TbRGG1, TbRGG2, and MRP1 onto mt transcripts, qualifying MRB8170 and MRB4160 as crucial players in coordinating the cross talk between MRB1 and other mtRNA processing complexes in *T. brucei*.

## DISCUSSION

In order to define the roles of MRB8170 and MRB4160 in RNA editing and/or processing *in vivo*, we captured their RNA-binding footprints using iCLAP. MRB8170 was shown to bind all three classes of maxicircle mRNAs, while MRB4160 was restricted to pan-edited and minimally edited transcripts. Thus, MRB8170 emerged as the more active paralog, which is consistent with the stronger phenotype caused by its depletion (24). Furthermore, while both proteins preferentially bind pan-edited mRNAs, there is a striking positive correlation between the amount of binding to a given transcript and the extent of editing. Moreover, the genomic snapshots of MRB8170/MRB4160 iCLAP tags demonstrated that both proteins bind over the entire length of preedited mRNAs, seemingly as a hallmark of their participation in this process. In support of this hypothesis, MRB8170 and MRB4160 iCLAP tags are absent on preedited versions of *ND3* and *CR4* mRNAs, which are transcribed but not edited in the procyclic stage examined here (30, 32). The iCLAP data are therefore compatible with a binding of both proteins to preedited transcripts as a prerequisite for editing. The sharp decrease in the abundance of fully edited versions of pan-edited and minimally edited transcripts upon simultaneous depletion of MRB8170 and MRB4160 further supports this argument (24). Combined with previous findings, our data show that MRB8170 and/or MRB4160 is indispensable for the editing of both pan-edited and minimally edited transcripts (24, 37).

In contrast to its binding to preedited forms of pan-edited and minimally edited mRNAs, the binding of MRB8170 to never-edited transcripts showed an inverse relationship with their abundance. This observation might indirectly explain the accumulation of never-edited transcripts in flagellates depleted of MRB8170 and MRB4160 (24). The negative impact of MRB8170 binding on the abundance of never-edited transcripts is intriguing and may also involve its interaction partner TbRGG2, which was reported to destabilize never-edited transcripts (27, 37, 38). Among all tested maxicircle transcripts, three showed an unexpected behavior. Although preedited *ND7* and *ND8* were extensively bound by both MRB8170 and MRB4160, suggesting their efficient editing, the low abundance of fully edited versions in the procyclic stage suggests that additional proteins are involved in their regulation (30, 32). Several subunits of the MRB1 core complex represent suitable candidates for such a function, as they were reported to affect a subset of pan-edited transcripts (18). Also, the RNA editing helicase 2 (REH2)-associated subcomplex was recently shown to act in parallel to MRB8170 and MRB4160 (17). Moreover, the stage-specific regulation of *MURF1* mRNA guided by its poly(A/U) tail implicates the polyadenylation mediator complex (PAMC) as yet another player in maintaining the steady-state level of some maxicircle transcripts (9, 11, 40).

We provide evidence that MRB8170 and MRB4160 are a nexus between RNA editing and other processing steps. Both proteins satisfy the following requirements to be considered for such a role: (i) they interact with MRB1 core proteins, and their simultaneous depletion compromises the overall integrity of MRB1; (ii) they share a number of RNase-resistant interacting partners outside MRB1 that belong to other processing complexes; (iii) they bind both preedited and edited mRNAs; and (iv) their simultaneous depletion affects the steady-state abundance of all three categories of maxicircle mRNAs. Below, we elaborate on the basis for these conclusions, ultimately proposing a model of how MRB1 functions in shaping the mt transcriptome.

In agreement with a previous study, our data show that the MRB1 core component GAP1, as well as the accessory protein TbRGG2, is a stable interacting partner of MRB8170 and MRB4160 (24). Moreover, our analyses further support the idea that MRP1, TbRGG1, and Nudix hydrolase are associated with both proteins. To seek further support for this hypothesis, *in vivo* mt mRNA interactome pulldown experiments were carried out in the presence and absence of both MRB8170 and MRB4160. In the latter samples, TbRGG1, TbRGG2, MRP1, and Nudix hydrolase showed a substantial reduction in poly(A) RNA binding. These results allow us to postulate that TbRGG1, TbRGG2, MRP1, and Nudix hydrolase require the assistance of MRB8170 and MRB4160 to bind mRNA. The *in vivo* data were further supported by the observation that addition of recombinant MRB8170 was sufficient to enhance poly(A) RNA binding of TbRGG2 *in vitro*. Taken together, we provide strong evidence that MRB8170 and MRB4160 enhance the activity of other mt RBPs, presumably by attracting or stabilizing them to transcripts already decorated by one or both of these paralogs.

**FIG 7:**
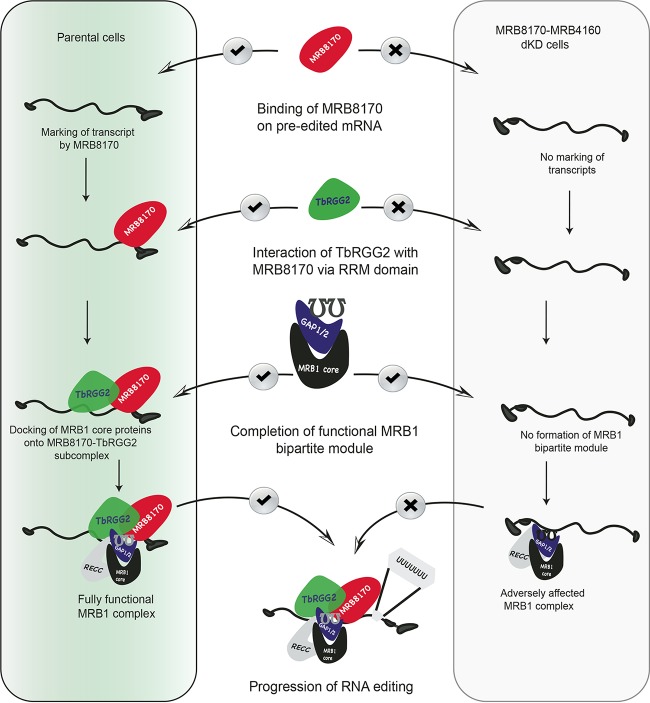
Schematic depiction of the formation of MRB1 bipartite modules in wild-type and MRB8170/MRB4160-depleted cells. In wild-type *T. brucei* (green), the preferential binding of MRB8170 and/or MRB4160 to preedited mRNAs marks the initiation of RNA editing, followed by their binding of TbRGG2 via its RRM domain. Subsequently, the gRNA-loaded MRB1 core proteins dock into the MRB8170-TbRGG2 (or MRB4160-TbRGG2 [data not shown]) subcomplex, eventually completing the MRB1 complex. In contrast, in MRB8170/MRB4160-depleted cells (light gray), MRB1 complex fails to come together, consequently undermining the RNA editing process.

Based on the above results and previous studies (27), we propose a scenario for the regulatory interplay between MRB8170 and TbRGG2 in which the N-terminal RNA recognition motif (RRM) domain of TbRGG2 mediates its interaction with MRB8170 and/or MRB4160 (Fig. 7). This interaction frees the TbRGG2 C-terminal G-rich domain, which was previously sequestered by interaction with the RRM domain, to bind RNA (27, 37). This hypothesized interplay between MRB8170 and TbRGG2 brings a new perspective on how MRB1 is involved in RNA editing. In a model that attempts to integrate the iCLAP data with our *in vivo* and *in vitro* results, the preferential binding of MRB8170 and/or MRB4160 onto preedited mRNAs marks the initiation of RNA editing, followed by binding of TbRGG2 via its RRM domain (Fig. 7). Subsequently, the gRNA-loaded MRB1 core proteins dock into the MRB8170-TbRGG2 (or MRB4160-TbRGG2) subcomplex (also known as the RNA editing mediator complex [REMC]), bringing the MRB1 complex together (19). In the absence of MRB8170 and MRB4160, the bipartite module fails to form, leading to a general reduction in the abundance of fully edited transcripts and an eventual impact on parasite fitness.

## MATERIALS AND METHODS

### iCLAP protocol.

For a single purification, 500 ml of cells expressing mTAP-tagged MRB8170 or MRB4160 was harvested after 2 days of induction. For *in vivo* UV-cross-linking experiments, cells were washed once and then resuspended in 25 ml of ice-cold phosphate-buffered saline (PBS) and placed in a petri dish 5 cm from the light source for UV irradiation (0.8 J/cm^2^ at 254 nm for iCLAP library preparation) in a Stratalinker 1800 machine (Stratagene). After a quick spin, the cells were snap-frozen in liquid nitrogen and stored at −80°C until further use. Cell pellets (~1.0 to 1.5 g [dry weight]) were resuspended in 5 ml of lysis buffer (50 mM Tris, pH 7.6, 1.5 mM MgCl_2_, 10% glycerol, 250 mM NaCl, 2.5 mM β-mercaptoethanol, 0.5% NP-40, 0.1% SDS) containing Complete EDTA-free protease inhibitor cocktail for 10 min on ice. The cell suspension was lysed and spun down by centrifugation (20 min at 20,000 × *g* at 4°C). The supernatant was treated with Turbo DNase (Life Technologies) and RNase I at 37°C for 3 min and then incubated on ice for 3 min as recommended in the published protocol (26). The recovered RNA was used to prepare iCLAP libraries using a previously published protocol (26). The specificity and efficiency of the affinity purification were confirmed by SDS-PAGE and Western blot analysis using anti-His antibody to detect the mTAP-tagged MRB8170 and MRB4160, which also bear this epitope.

### Next-generation sequencing and computational analysis.

MRB8170, MRB4160, and control (UV-cross-linked parental cells) iCLAP cDNA libraries were sequenced using Illumina Hi-Seq 2000 (single-end sequencing, 75-nt length). Raw reads were trimmed of 3′ adaptor sequences (Tag cleaner version 0.16), and PCR duplicates were collapsed (Fastx collapser version 0.13). The remaining reads were ~30 to 50 nt long. The reads were divided into individual replicates using 4-nt experimental barcodes and mapped first onto preedited (GenBank sequence accession no. M94286) and then to fully edited (39) sequences using Bowtie (Bowtie2 version 0.2) with “very sensitive” preset and a mismatch penalty tightened to 1. More details are in Text S1 in the supplemental material.

10.1128/mBio.02288-16.9 TEXT S1 Extended experimental procedures. Download TEXT S1, DOCX file, 0.05 MB. Copyright © 2017 Dixit et al. 2017 Dixit et al. This content is distributed under the terms of the Creative Commons Attribution 4.0 International license.

### Accession number(s).

All the iCLAP sequences are available at ArrayExpress with accession number E-MTAB-4934.
