# Supplementary material for: Differential Binding of Mitochondrial Transcripts by MRB8170 and MRB4160 Regulates Distinct Editing Fates of Mitochondrial mRNA in Trypanosomes
Source: mBio. 2017 Jan 31;8(1):e02288-16. doi: 10.1128/mBio.02288-16 (PMC5285507; doi:10.1128/mBio.02288-16)
Supplement: TABLE S1 [file mbo001173170st1.pdf]

Subcomplex	Name	Alias	Domains	TriTrypDB #
Core	GAP1	GRBC2		Tb927.2.3800
Core	GAP2	GRBC1		Tb927.7.2570
Core	MRB3010	GRBC6	RPS2	Tb927.5.3010
Core	MRB5390	GRBC4		Tb11.02.5390
Core	MRB8620	GRBC3		Tb927.11.16860
Core	MRB11870	GRBC5	pentein	Tb927.10.11870
Core	MRB0880	GRBC7		Tb927.11.9140
Core	RBP30	none	RRM	Tb927.5.2100
Core	none	none		Tb927.9.1420
Core	none	none		Tb927.10.10120
TbRGG2	TbRGG2	none	RRM, RGG	Tb927.10.10830
TbRGG2	MRB1860	REMC2		Tb927.2.1860
TbRGG2	MRB4160	REMC5		Tb927.4.4160
TbRGG2	MRB800	REMC3		Tb927.7.800
TbRGG2	MRB8170	REMC5A		Tb927.8.8170
TbRGG2	MRB8180	REMC4		Tb927.8.8180
TbRGG2	PhyH	none	PhyH	Tb927.9.7260
Unknown	MRB10130	REMC1	ARM/HEAT	Tb927.10.10130

Please refer to Read et al. 2016 (Ref 6) for more details

TABLE S1. MRB1 complex Subunits
Listed are the proteins which were detected by various labs to comprise the MRB1 complex
