# Supplementary material for: Differential Binding of Mitochondrial Transcripts by MRB8170 and MRB4160 Regulates Distinct Editing Fates of Mitochondrial mRNA in Trypanosomes
Source: mBio. 2017 Jan 31;8(1):e02288-16. doi: 10.1128/mBio.02288-16 (PMC5285507; doi:10.1128/mBio.02288-16)
Supplement: FIG S1 [file mbo001173170sf1.pdf]

SUPPLEMENTARY FIGURES

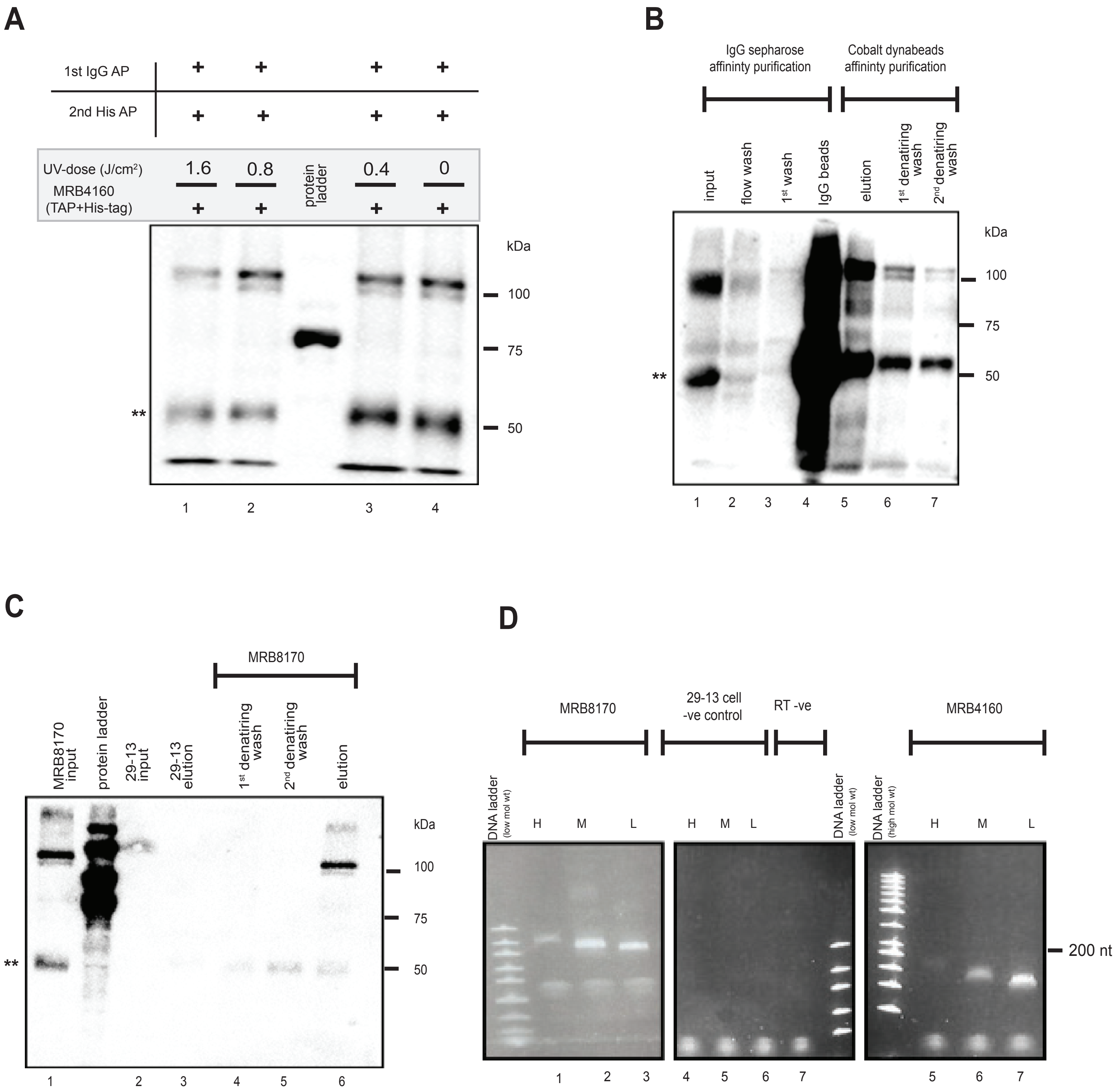

Figure S1.

Figure S1. MRB8170 and MRB4160 iCLAP characterization

(A) Specificity and efficiency of affinity purified, UV crosslinked TAP-tagged MRB4160. The co-purified UV cross-linked RNA-MRB4160 complex after two step affinity purification was confirmed by SDS-PAGE and Western blot analysis. The high-RNAse I treated samples from four different UV doses (1.6, 0.8, 0.4, and 0 J/cm^2) were separately resolved using SDS-PAGE and transferred onto a nitrocellulose membrane, which was probed using α-His antibody, recognizing an epitope on the modified TAP-tag. MRB4160 was detected at its ~100 kDa size (lanes 1-4).
**Partially degraded MRB4160 (modified TAP-tag) protein.

(B) Specificity and efficiency of affinity purified, UV crosslinked TAP-tagged MRB8170. The co-purified low RNAse-I treated 0.8 J/cm^2 UV crosslinked MRB8170 with modified TAP-tag was followed throughout two steps of affinity purification, fractions of which are indicated at the top. The membrane was probed as in (A). MRB8170 migrated at ~100 kDa (lanes 1-7). **Partially degraded MRB8170 (modified TAP-tag) protein.

(C) Specificity and efficiency of affinity-purified, non-UV crosslinked TAP-tagged MRB8170. The non-UV cross-linked TAP-tagged MRB8170 and control parental cell lines were processed and assayed as in (A) and (B). MRB8170 migrated at the expected ~100 kDa (lane 1, and 6), while there was no signal in the control (lane 3). Labeling as in (B).

(D) TBE-6% urea gel of the amplified iCLAP sequencing libraries from TAP-tagged MRB8170 and MRB4160, plus parental cell lines. The cDNA was size fractionated and amplified separately to reduce any size biases during PCR. Three sizes were cut out and PCR-amplified, indicated as high (H) (150-300 nucleotides, lanes 1,4 and 8), medium (M) (80-150 nucleotides, lanes 2, 5 and 9) and low (L) (60-80 nucleotides, lanes 3, 6 and 10). The H and M nucleotide long libraries were submitted for next-generation sequencing. RT –ve, reverse transcriptase control (lane 7); MRB4160, iCLAP libraries from UV crosslinked modified TAP-tag MRB4160 (lanes 5, 6 and 7); MRB8170, iCLAP libraries from UV crosslinked modified TAP-tagged MRB8170 (lanes 1, 2 and 3); 29-13 negative control (lanes 4, 5 and 6); iCLAP libraries from UV crosslinked parental 29-13 strain (mock affinity purification). After the removal of adapter sequence the resulting iCLAP tags were ~30 to 50 bp in size.
