# Supplementary material for: Differential Binding of Mitochondrial Transcripts by MRB8170 and MRB4160 Regulates Distinct Editing Fates of Mitochondrial mRNA in Trypanosomes
Source: mBio. 2017 Jan 31;8(1):e02288-16. doi: 10.1128/mBio.02288-16 (PMC5285507; doi:10.1128/mBio.02288-16)
Supplement: FIG S2 [file mbo001173170sf2.pdf]

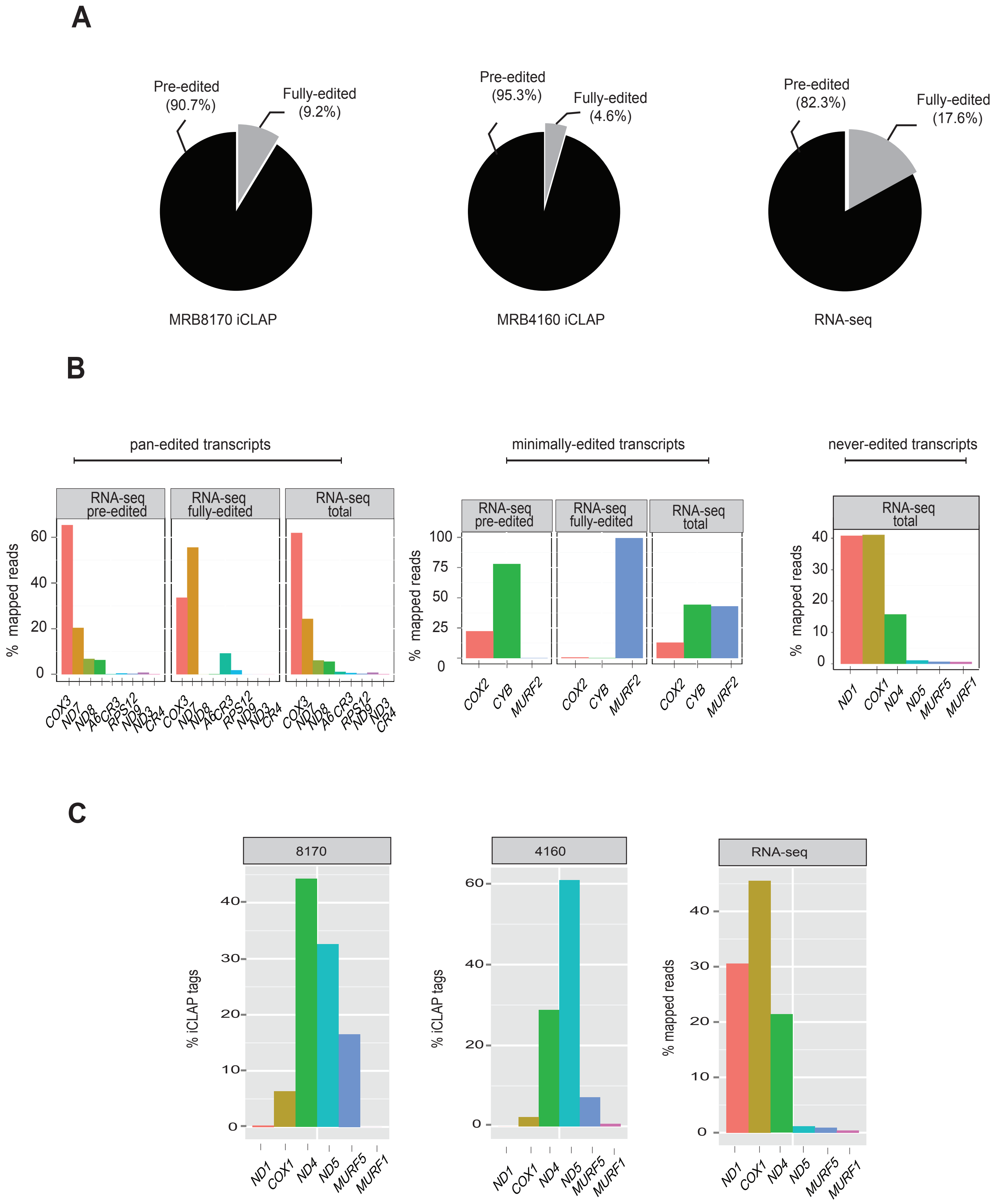
A
Pre-edited (90.7%)
Fully-edited (9.2%)
MRB8170 iCLAP
Pre-edited (95.3%)
Fully-edited (4.6%)
MRB4160 iCLAP
Pre-edited (82.3%)
Fully-edited (17.6%)
RNA-seq
B
pan-edited transcripts
minimally-edited transcripts
never-edited transcripts
RNA-seq pre-edited
RNA-seq fully-edited
RNA-seq total
% mapped reads
COX3
ND7
ND8
A6
CR3
RPS12
ND9
ND3
CR4
COX2
CYB
MURF2
ND1
COX1
ND4
ND5
MURF5
MURF1
C
8170
4160
RNA-seq
% iCLAP tags
% mapped reads

Figure S2.

Figure S2. iCLAP and RNA-seq binding to maxicircle

(A) Pie chart of uniquely mapped iCLAP tags (~30-50 nt long) and RNA-seq reads to the maxicircle genome. Percentage of MRB8170 iCLAP tags, MRB4160 iCLAP tags and RNA-seq reads to pre- and fully-edited maxicircle transcripts. Pre-edited, black; fully-edited, grey. Never-edited transcripts were excluded for the analyses. Our protocol successfully obtained ~30-50 nt long iCLAP tags, after the removal of the adaptor sequences. A drawback of the short read length of iCLAP tags is the resulting inability to quantitate the amount of reads originating from partially-edited mRNAs, therefore, in both cases, it is impossible to quantitate the amount of reads originating from partially-edited mRNAs, which creates a bias in the number of pre- and fully-edited iCLAP pie chart.

(B) Percentage of uniquely mapped RNA-seq reads to the three classes of maxicircle mRNAs. Three bar plots showing the percentage of uniquely mapped RNA-seq reads to pan-edited transcripts in the pre-edited and fully-edited regions, plus the total of the two (pre-edited and fully-edited), respectively, for each of the mt RNAs indicated on the x-axis.

(C) Normalized bar plot with respect to gene length of never-edited transcripts. Three bar plots show the normalized data (using DEX-seq) with respect to gene length of MRB8170 iCLAP tags, MRB4160 iCLAP tags, and RNA-seq reads mapped to never-edited transcripts of the mt mRNA are indicated on the x-axis.
