# Supplementary material for: Differential Binding of Mitochondrial Transcripts by MRB8170 and MRB4160 Regulates Distinct Editing Fates of Mitochondrial mRNA in Trypanosomes
Source: mBio. 2017 Jan 31;8(1):e02288-16. doi: 10.1128/mBio.02288-16 (PMC5285507; doi:10.1128/mBio.02288-16)
Supplement: FIG S3 [file mbo001173170sf3.pdf]

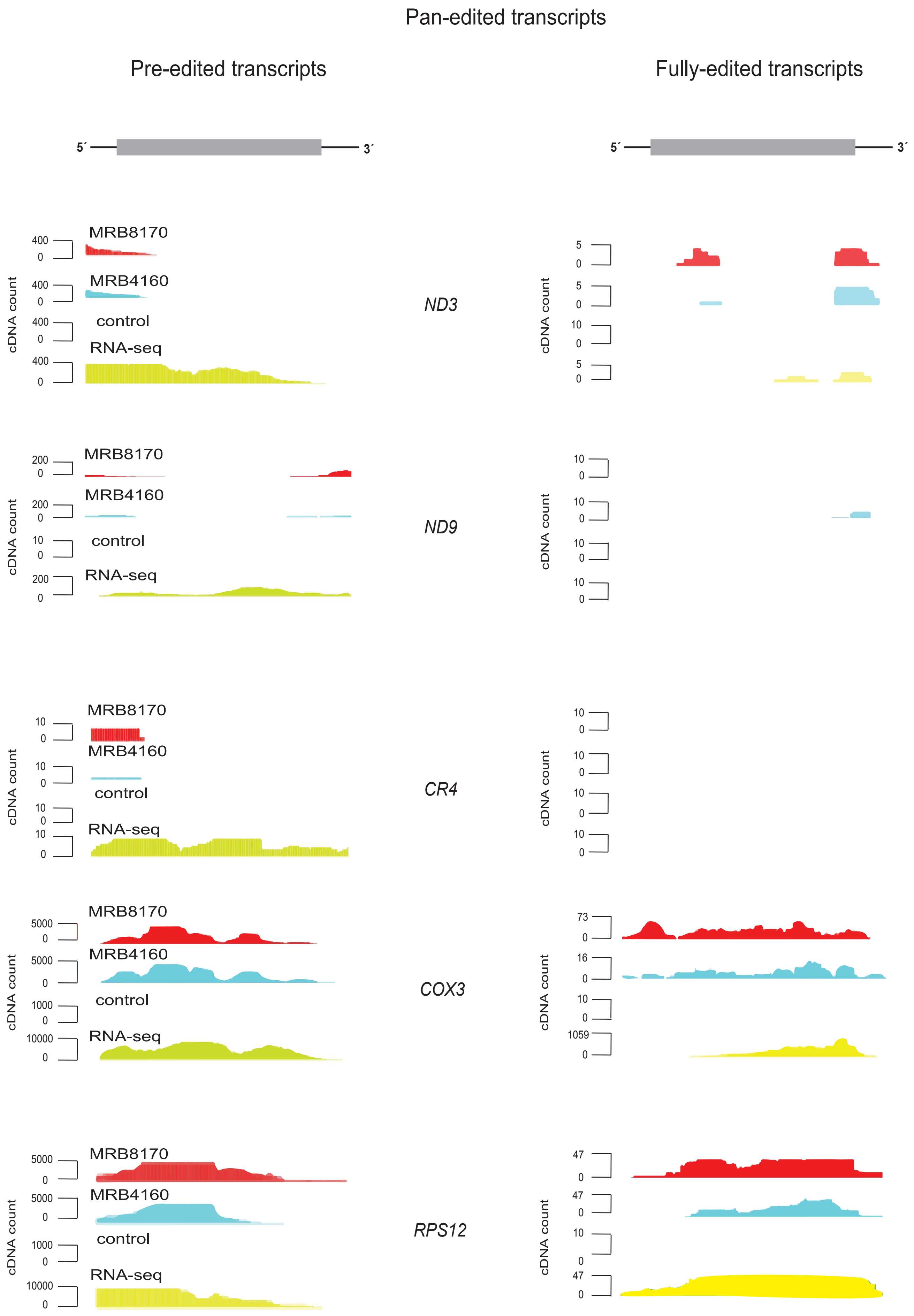
Pan-edited transcripts
Pre-edited transcripts
Fully-edited transcripts
5´
3´
ND3
ND9
CR4
COX3
RPS12
MRB8170
MRB4160
control
RNA-seq
cDNA count

Figure S3.

Figure S3. MRB8170 and MRB4160 binding to a subset of pan-edited transcripts (*ND3, ND9, CR4, COX3* and *RPS12*)

Genomic browser snapshot of mapped iCLAP-tags and RNA-seq reads to individual pan-edited transcripts, for which the pre-edited and fully-edited versions are shown separately. The unique cDNA count is depicted on the y-axis and the mapped tag position along a given transcript is shown on the x-axis. MRB8170 iCLAP-tags are in red, MRB4160 iCLAP-tags in blue, control iCLAP-tags in black and RNA-seq reads in yellow. *ND3, ND9* and *CR4* lacks significant iCLAP tags from both MRB8170 and MRB4160 to pre-edited sequence. The other 6 pan-edited mRNAs (*COX3, RPS12, CR3, ND8, ND7,* and *A6*) shared varying order of iCLAP tags from MRB8170 and MRB4160, positively correlating with their editing extent.
