# Supplementary material for: Differential Binding of Mitochondrial Transcripts by MRB8170 and MRB4160 Regulates Distinct Editing Fates of Mitochondrial mRNA in Trypanosomes
Source: mBio. 2017 Jan 31;8(1):e02288-16. doi: 10.1128/mBio.02288-16 (PMC5285507; doi:10.1128/mBio.02288-16)
Supplement: FIG S4 [file mbo001173170sf4.pdf]

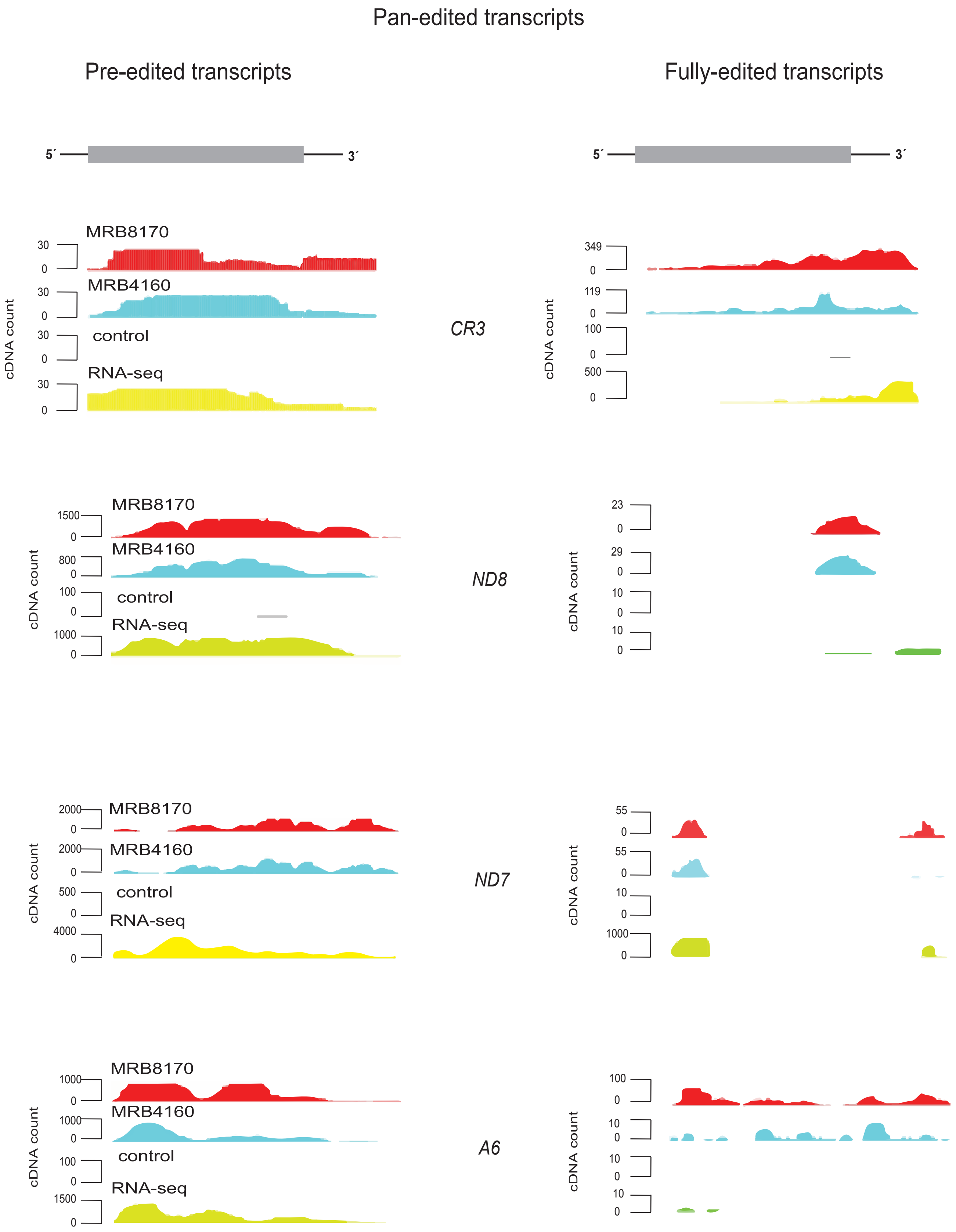

Figure S4. MRB8170 and MRB4160 binding to a subset of pan-edited transcripts (*CR3, ND8, ND7,* and *A6*)
Labeling as in Figure (S3)

Figure S4.
