# Supplementary material for: Differential Binding of Mitochondrial Transcripts by MRB8170 and MRB4160 Regulates Distinct Editing Fates of Mitochondrial mRNA in Trypanosomes
Source: mBio. 2017 Jan 31;8(1):e02288-16. doi: 10.1128/mBio.02288-16 (PMC5285507; doi:10.1128/mBio.02288-16)
Supplement: FIG S5 [file mbo001173170sf5.pdf]

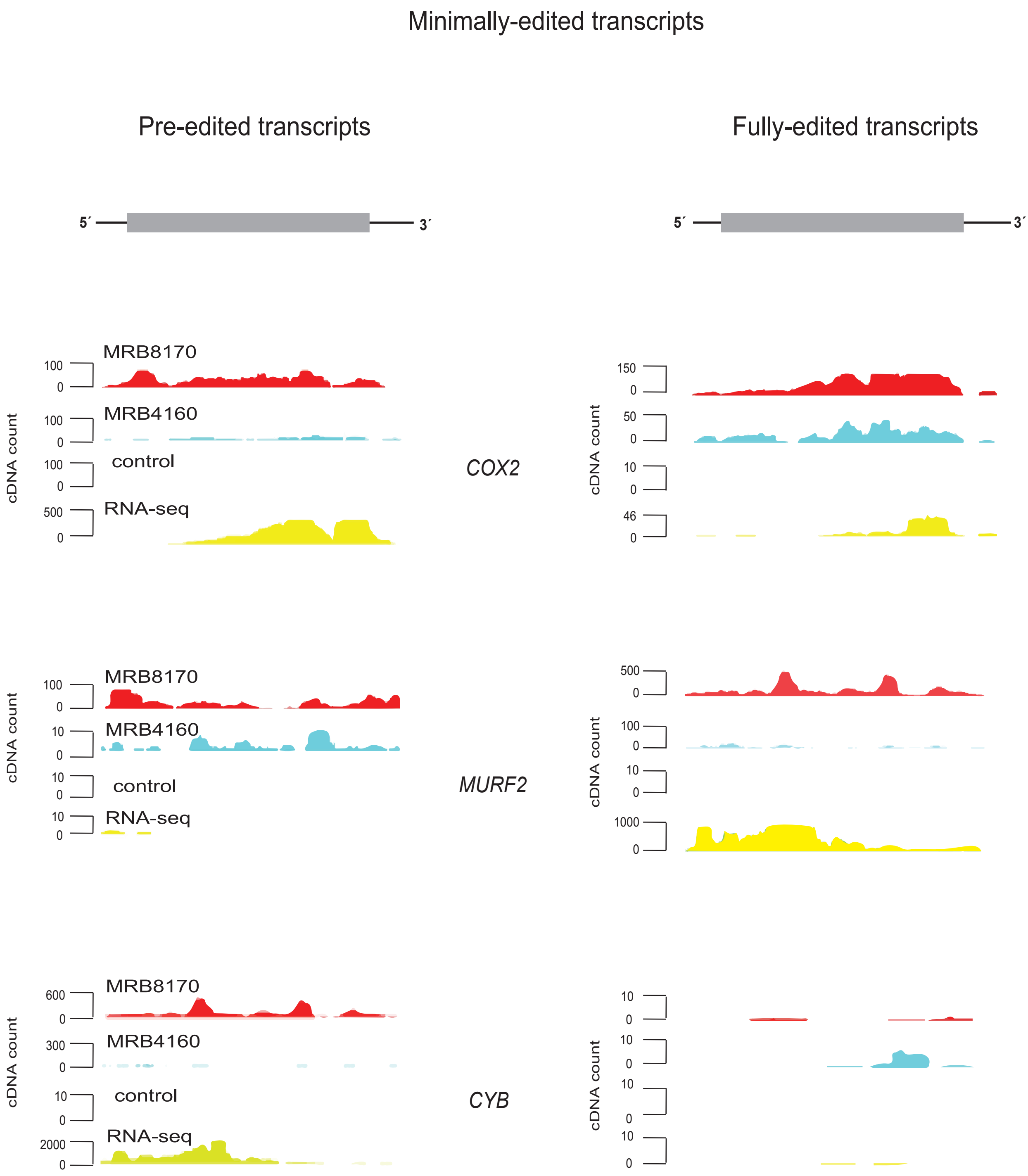

Figure S5. MRB8170 and MRB4160 binding to minimally-edited transcripts

Genomic browser snapshot of mapped iCLAP-tags and RNA-seq reads to *COX2, MURF2* and *CYB* transcripts. Labeled as in Figure S3. The genome browser snapshots depicted the binding of proteins over the entire pre-edited *COX2, MURF2* and *CYB* transcripts.

Figure S5.
