# Supplementary material for: Differential Binding of Mitochondrial Transcripts by MRB8170 and MRB4160 Regulates Distinct Editing Fates of Mitochondrial mRNA in Trypanosomes
Source: mBio. 2017 Jan 31;8(1):e02288-16. doi: 10.1128/mBio.02288-16 (PMC5285507; doi:10.1128/mBio.02288-16)
Supplement: FIG S6 [file mbo001173170sf6.pdf]

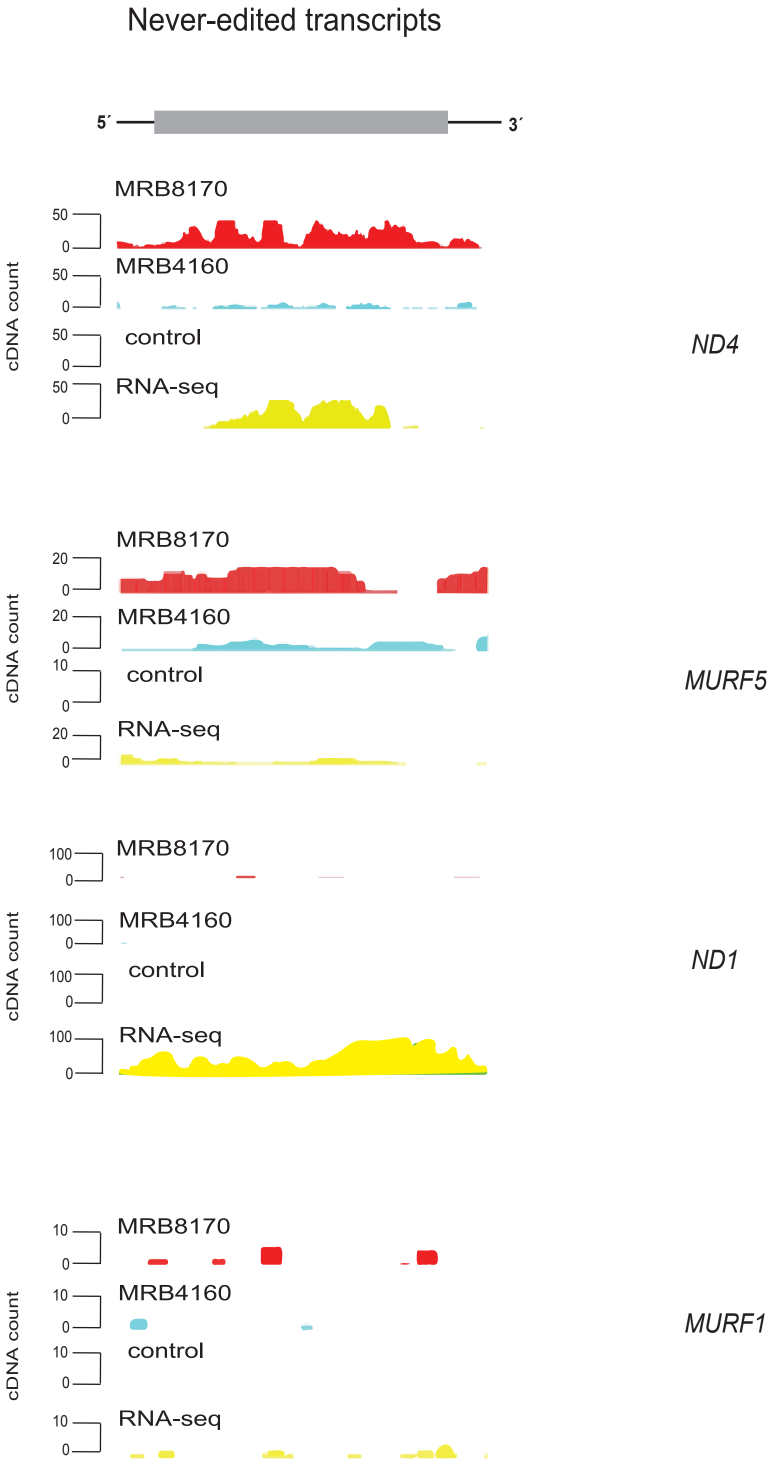

Figure S6. MRB8170 and MRB4160 preferential binding to never-edited transcripts

Genomic browser snapshot of mapped iCLAP-tags and RNA-seq reads to *ND4, MURF5, ND1,* and *MURF1* transcripts. Labeled as in Figure S3. The other two never-edited transcripts (*ND4* and *COX1*), are part of the main figure (Figure 4B). *ND1* and *COX1* lack significant iCLAP tags, whereas both shows significant RNA-seq reads.

Figure S6.
