# Supplementary material for: Differential Binding of Mitochondrial Transcripts by MRB8170 and MRB4160 Regulates Distinct Editing Fates of Mitochondrial mRNA in Trypanosomes
Source: mBio. 2017 Jan 31;8(1):e02288-16. doi: 10.1128/mBio.02288-16 (PMC5285507; doi:10.1128/mBio.02288-16)
Supplement: FIG S7 [file mbo001173170sf7.pdf]

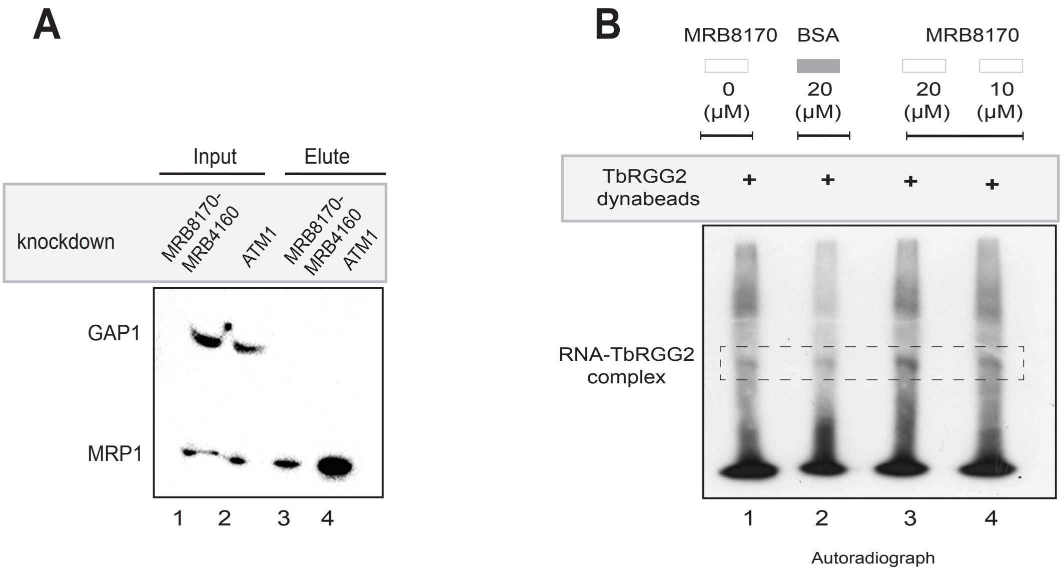

Figure S7. MRB8170- and MRB4160-associated interactome

(A) Western blot analysis of total extracts (lanes 1 and 2) and oligo-dT eluates (lanes 3 and 4) from MRB8170/MRB4160 and ATM1 RNAi-induced knockdown cells. GAP1 and MRP1 were detected in parallel using the same PVDF membrane to serve as a loading control for the oligo-dT experiment.

(B) *In vitro* CLIP assay from Figure 5 (C). The ^{32}P labelled RNA from immunoprecipitated TbRGG2-RNA complex was visualized by autoradiography (Autoradiograph; lanes 1-4). The untrimmed autoradiography film is shown.

Figure S7.
