# Supplementary material for: Differential Binding of Mitochondrial Transcripts by MRB8170 and MRB4160 Regulates Distinct Editing Fates of Mitochondrial mRNA in Trypanosomes
Source: mBio. 2017 Jan 31;8(1):e02288-16. doi: 10.1128/mBio.02288-16 (PMC5285507; doi:10.1128/mBio.02288-16)
Supplement: TEXT S1 [file mbo001173170s1.docx]

**Extended Materials and Methods**

***T. brucei* culture conditions and generation of cell lines**

Previously constructed cell lines procyclic form (PF) *T. brucei* (strain 29-13) harboring MRB8170 and MRB4160 with mTAP tags (1), plus those for simultaneously inducible RNAi silencing of MRB8170 and MRB4160 or ATM1 were used as previously described (1, 2). RNAi was induced by the addition of 1 µg/ml tetracycline to culture media. Cell densities were measured by using a Z2 cell counter (Beckman Coulter Inc) and were maintained in the exponential mid-log growth phase at 27°C with constant shaking.

**Quantitative real-time PCR analysis of knockdown cells**

MRB8170/MRB4160 (1) and ATM1 (2) knockdown cells were harvested after 48 hr of RNAi induction and RNA was isolated and processed for reverse transcription into cDNA. The cDNA was used for quantitative (q) PCR analysis with primers recognizing specific mt mRNA sequences. The raw qPCR data were analyzed using Pfaffl method (3), with 18S rRNA as an internal control. The relative abundances of the examined transcripts were compared between both cell lines. All qPCR protocols were performed in triplicate, and both cell lines were processed in parallel. The primers and qPCR conditions were as previously described (4).

**SDS-PAGE and Western blot analysis**

Primary antibodies used in this study were: α-MRB8170 (1:1,000) (1), α-GAP1 (1:1,000) (5), α-MRP1 (1:2,000) (6), α-TbRGG1 (1:500) (7), α-TbRGG2 (1:1,000) (produced in this study against recombinant TbRGG2), α-REL1 (1:100), α-Nudix hydrolase (1:1,000) (produced in this study against recombinant Nudix hydrolase, Tb11.01.7290), and α-His (1:1,000) (Invitrogen).

**RNA immunoprecipitation and quantitative real-time PCR**

MRB8170-mTAP, MRB4160-mTAP and parental cell lines (mock) were resuspended in RIP lysis buffer (50 mM Tris pH 7.5, 250 mM NaCl, 1 mM DTT, 10% glycerol, 0.5% NP-40) containing cOmplete EDTA-free Protease Inhibitor Cocktail (Roche) and RNaseOUT (100 units; Life Technologies). The supernatant was Turbo DNase treated (Life Technologies) and subjected to immunoprecipitation using IgG-Sepharose beads (GE Healthcare) for 2 hr at 4°C. Ten percent of the supernatant was taken as input to generate cDNA for normalizing the respective immunoprecipitated samples. After immunoprecipitation, the beads were washed with RIP wash buffer (50 mM Tris pH7.5, 1 M NaCl, 0.5% NP40, and 0.1% SDS) three times before phenol extraction of RNA. RNA obtained from the supernatant (input) and eluate (output) was transcribed into cDNA and further analyzed by qPCR (Light Cycler, Roche). The relative ratios were calculated for each immunoprecipitated sample normalized against their respective input. Construction of cDNA was done using the QuantiTect Reverse Transcription Kit (Qiagen). Previously designed primers that anneal to the specified maxicircle mRNA sequences were used in qPCR (4). The PCR conditions were 95°C for 10 min, followed by 40 cycles [95°C for 15 sec, 60°C for 1 min].

**Rapid tandem affinity purification and protein analysis**

A volume of 250 ml of cells with MRB8170 or MRB4160 bearing the modified TAP-tag were harvested 2 days after tetracycline induction and resuspended in RAP lysis buffer (25 mM Tris pH 7.5, 100 mM NaCl, 1 mM DTT, 0.5% NP-40) containing the cOmplete EDTA-free Protease Inhibitor Cocktail (Roche). The lysate was pre-cleared and RNase l and Turbo DNase were added to the supernatant. IgG-coated Dynabeads were also blocked with 1% BSA and then equilibrated with lysis buffer as described elsewhere (8). Subsequently, the supernatants were loaded onto IgG Dynabeads for 1 hr at 4°C. The beads were washed three times with RAP wash buffer (25 mM Tris pH 7.5, 300 mM NaCl, 1 mM DTT, 0.1% NP-40), and the elution was carried out using AcTEV protease (Invitrogen). The eluate was resolved by SDS-PAGE, blotted onto a PVDF membrane and subsequently probed for TbRGG2, GAP1, MRP1, Nudix hydrolase, and TbRGG1 using the antibodies listed above.

***In vivo* UV crosslinking and oligo-dT pulldown**

Two liters of MRB8170/MRB4160 double knockdown and ATM1 single knockdown cells were harvested after 48 hr of RNAi-induction and *in vivo* UV crosslinked using similar conditions as those used in the iCLAP protocol. Hypotonically purified mt vesicles obtained from freshly UV crosslinked cells were resuspended in an oligo lysis buffer (20 mM Tris pH 7.4, 500 mM LiCl, 0.5% LiDS, 1 mM EDTA and 5 mM DTT) supplemented with cOmplete EDTA-free Protease Inhibitor Cocktail and RNaseOUT for 15 min on ice (9, 10). The lysates were pre-cleared and protein concentration was determined by Nanodrop spectrophotometer. Supernatants of equal protein concentration were loaded onto pre-equilibrated oligo-(dT)_25_ beads (Life Technologies) with an oligo lysis buffer for 2 hr at 4°C. Oligo (dT)_25_ beads with captured mt mRNA-protein complex went through three washing steps, starting with wash buffer 1 (20 mM Tris pH 7.4, 500 mM LiCl, 0.1% LiDS, 1 mM EDTA, 5 mM DTT and 8 M urea), followed by wash buffers 2 (20 mM Tris pH 7.4, 500 mM LiCl, 0.5% LiDS, 1 mM EDTA and 5 mM DTT) and 3 (20 mM Tris pH 7.4, 200 mM LiCl, 1 mM EDTA and 5 mM DTT). Finally, the beads were incubated with LDS sample buffer (Life Technologies) and incubated for 10 min at 80°C. Protein eluates were separated using NuPAGE Novex 4-12% Bis-Tris gels (Invitrogen) and blotted onto a PVDF membrane, which was probed with antibodies against DRBD18 (1:1,000) (11), Nudix hydrolase, TbRGG2, MRP1, TbRGG1, REL1 and GAP1 using the aforementioned dilutions.

**iCLAP protocol**

For a single purification, 500 ml of cells expressing mTAP-tagged MRB8170 or MRB4160 were harvested after 2 days of induction. For *in vivo* UV crosslinking experiments, cells were washed once and then resuspended in 25 ml of ice-cold PBS and placed in a Petri dish 5 cm from the light source for UV irradiation (0.8 J/cm^2^ at 254 nm for iCLAP library preparation) in a Stratalinker 1800 machine (Stratagene). After a quick spin, the cells were snap-frozen in liquid nitrogen and stored at -80°C until further use. Cell pellets (~1.0-1.5 g dry weight) were resuspended in 5 ml of lysis buffer (50 mM Tris pH 7.6, 1.5 mM MgCl_2_, 10% glycerol, 250 mM NaCl, 2.5 mMβ-mercaptoethanol, 0.5% NP-40, 0.1% SDS) containing cOmplete EDTA-free Protease Inhibitor Cocktail for 10 min on ice. The cell suspension was lysed and spun down by centrifugation (20 min at 20,000 g at 4°C). The supernatant was treated with Turbo DNase (Life Technologies) and RNase I at 37°C for 3 min and then incubated on ice for 3 min as recommended in the published protocol (9). Afterwards, 5 ml of lysis buffer was added to the supernatant to the final volume of 10 ml. Next, this extract was loaded with 250 µl of equilibrated IgG sepharose beads for 2 hr at 4°C. After the incubation, three washes were performed with 3 ml of wash buffer (50 mM Tris pH 7.6, 500 mM NaCl, 2.5 mMβ-mercaptoethanol, 0.5% NP-40, 0.1% SDS) and three washes in TEV cleavage buffer (50 mM Tris pH 7.6, 100 mM NaCl, 2.5 mMβ-mercaptoethanol). Finally, the beads were resuspended in 600 µl of TEV cleavage buffer and incubated for 2 hr at 18°C with 10 µg of GST-TEV protease. The suspension was subsequently transferred into Micro Bio-Spin columns (BioRad) and centrifuged to collect the TEV eluates. Guanidine-HCL (0.4 g) was dissolved in 500 µl of TEV eluates (6 M final concentration), and NaCl and imidazole were added to final concentrations of 250 mM and 10 mM, respectively. Samples were added to 100 µl of pre-equilibrated His-Tag Isolation Dynabeads (Invitrogen) and agitated for 2 hr at 4 °C. Beads were washed twice with 1 ml of wash buffer (6 M guanidine-HCl, 50 mM Tris pH 7.6, 300 mM NaCl, 0.1 NP-40, and 10 mM imidazole). Afterwards, RNA was 5’-end labeled on bead with [γ-^32^P]-ATP and subsequently the 3’-end was ligated to an adaptor oligonucleotide. The RNA-protein complexes were separated by SDS-PAGE, transferred onto a nitrocellulose membrane and visualized by autoradiography. The areas corresponding to MRB8170- or MRB4160-RNA complexes were cut out and the bound RNA was released from the membrane by proteinase K treatment followed by phenol/chloroform extraction. The recovered RNA was used to prepare iCLAP libraries using a previously published protocol (9). The specificity and efficiency of the affinity purification was confirmed by SDS-PAGE and Western blot analysis using α-His antibody to detect the mTAP-tagged MRB8170 and MRB4160, which also bear this epitope.

**Computational analysis of Next Generation Sequencing data**

MRB8170, MRB4160 and control (UV-crosslinked parental cells) iCLAP cDNA libraries were sequenced using Illumina Hi-Seq 2000 (single-end sequencing, 75-nt length). Raw reads were trimmed of 3’ adaptor sequences (Tag cleaner version 0.16) and PCR duplicates were collapsed (Fastx collapser version 0.13). The remaining reads were ~30 to 50 nt long. The reads were divided into individual replicates using 4-nt experimental barcodes and mapped first onto pre-edited (GenBank M94286) and then to fully edited (12) sequences using Bowtie (Bowtie2 version 0.2) with ’very sensitive’ pre-set and a mismatch penalty tightened to 1. End-to-end mappings were generated with a set of arguments favoring insertions/deletions in overall strict alignment and minimizing heuristics (--rfg 10,10 --rdg 10,10 --mp 18 --end-to-end -D 20 -R 3 -N 0 -L 10 -i S, 1,0.50 -x). All end-to-end mappings were depleted of homopolymeric A and T reads, which otherwise inflated low complexity T-rich regions. Finally, individual mappings were split based on read orientation towards the reference sequence (Samtools version 1.12a). The random barcode allowed us to discriminate PCR duplicates from unique cDNAs that started at the same nucleotide, but derived from individual co-purified RNA molecules. Hence, random barcodes with more than one identical nucleotide were excluded, and only unique cDNAs were kept for further analyses. The first nucleotide upstream of the mapped cDNA was defined as the “crosslink nucleotide”, and all unique cDNA at this position were assigned as “cDNA count” (13). For all subsequent analyses, replicates were merged into one iCLAP dataset. In total, we obtained 191,683 uniquely aligned iCLAP tags for MRB8170 (137,365 and 54,318 for replicates 1 and 2, respectively) and 100,313 for MRB4160 (80,124 and 20,189). The control library from UV-crosslinked parental cells contained 483 uniquely mapping iCLAP tags (325 and 158 for replicates 1 and 2, respectively).

Bioinformatics analyses of two biological replicates from a recently published *T. brucei* RNA-seq study (11) were performed in a similar way as for the iCLAP data. The relevant RNA-seq reads were mapped against all forms of maxicircle transcripts with similar parameters as was done for iCLAP tags. The data visualization of iCLAP and RNA-seq data was done using Integrative Genomics Viewer (<https://www.broadinstitute.org/igv/>).

***In vitro* UV crosslinking and immunoprecipitation assay**

MRB8170/MRB4160 double knockdown cell lines were collected after 48 hr of RNAi induction and resuspended in lysis buffer (25 mM Tris pH 7.5, 100 mM NaCl, 1 mM DTT, 0.5% Triton X-100) containing cOmplete EDTA-free Protease Inhibitor Cocktail. The lysate was pre-cleared and RNaseOUT (100 units) was added. The supernatant was split into four tubes with equal protein concentration of supernatant and supplemented with 0, 10, and 20 µM of recombinant GST-MRB8170 (1) or 20 µM BSA. The 1 ml binding reaction was incubated for 30 min at 4°C before the samples were UV irradiated with 0.8 J/cm^2^ in a Stratalinker 1800 at 254 nm. The supernatant was treated with Turbo DNase and RNase I (Invitrogen) at 37°C for 3 min and then incubated on ice for 3 min. Next, the supernatant was incubated with α-TbRGG2 antibody-coated Protein A Dynabeads (Invitrogen) for 1 hr at 4°C followed by three washes with 1.5 ml of wash buffer (50 mM Tris pH 7.6, 500 mM NaCl, 2.5 mMβ-mercaptoethanol, 0.5% NP-40, 0.1% SDS). After stringent washes the bound RNA was 5’-end labeled with ^32^P directly on the beads. The RNA-protein complexes were separated by SDS-PAGE using NuPAGE Novex 4-12% Bis-Tris gels and transferred onto a nitrocellulose membrane. The RNA autoradiograph was developed after 8 hr of exposure onto Amersham Hyperfilm ECL (GE Healthcare) and the same membrane was probed using α-TbRGG2 antibody. In a parallel experiment, RNA was extracted after the stringent washes instead of running them on SDS-PAGE. The obtained RNA was reverse transcribed to cDNA and subsequently used for qPCR as described above.

**Statistical Analysis**

All graphs and statistical analyses were done in GraphPad Prism 5.00 (GraphPad, San Diego, CA, USA).

**References**

1. **Kafkova L**, **Ammerman ML**, **Faktorova D**, **Fisk JC**, **Zimmer SL**, **Sobotka R**, **Read LK**, **Lukes J**, **Hashimi H**. 2012. Functional characterization of two paralogs that are novel RNA binding proteins influencing mitochondrial transcripts of *Trypanosoma brucei*. RNA **18:** 1846–1861.

2. **Horáková E**, **Changmai P**, **Paris Z**, **Salmon D**, **Lukeš J**. 2015. Simultaneous depletion of ATM and Mdl rebalances cytosolic Fe-S cluster assembly but not heme import into the mitochondrion of *Trypanosoma brucei*. FEBS J **282:** 4157-4175.

3. **Pfaffl MW**. 2001. A new mathematical model for relative quantification in real-time RT-PCR. Nucleic Acids Res **29**:e45.

4. **Carnes J**, **Trotter JR**, **Ernst NL**, **Steinberg A**, **Stuart K**. 2005. An essential RNase III insertion editing endonuclease in *Trypanosoma brucei*. Proc Natl Acad Sci U S A **102**:16614–9.

5. **Hashimi H**, **Cicová Z**, **Novotná L**, **Wen Y-Z**, **Lukes J**. 2009. Kinetoplastid guide RNA biogenesis is dependent on subunits of the mitochondrial RNA binding complex 1 and mitochondrial RNA polymerase. RNA **15**:588–99.

6. **Schumacher MA**, **Karamooz E**, **Zíková A**, **Trantírek L**, **Lukeš J**. 2006. Crystal Structures of T. brucei MRP1/MRP2 Guide-RNA Binding Complex Reveal RNA Matchmaking Mechanism. Cell **126**:701–711.

7. **Hashimi H**, **Zíková A**, **Panigrahi AK**, **Stuart KD**, **Lukes J**. 2008. TbRGG1, an essential protein involved in kinetoplastid RNA metabolism that is associated with a novel multiprotein complex. RNA **14**:970–980.

8. **Oeffinger M**, **Wei KE**, **Rogers R**, **DeGrasse J a**, **Chait BT**, **Aitchison JD**, **Rout MP**. 2007. Comprehensive analysis of diverse ribonucleoprotein complexes. Nat Methods **4**:951–956.

9. **Huppertz I**, **Attig J**, **D’Ambrogio A**, **Easton LE**, **Sibley CR**, **Sugimoto Y**, **Tajnik M**, **König J**, **Ule J**. 2014. iCLIP: protein-RNA interactions at nucleotide resolution. Methods **65**:274–87.

10. **Castello A**, **Horos R**, **Strein C**, **Fischer B**, **Eichelbaum K**, **Steinmetz LM**, **Krijgsveld J**, **Hentze MW**. 2013. System-wide identification of RNA-binding proteins by interactome capture. Nat Protoc **8**:491–500.

11. **Lott K**, **Mukhopadhyay S**, **Li J**, **Wang J**, **Yao J**, **Sun Y**, **Qu J**, **Read LK**. 2015. Arginine methylation of DRBD18 differentially impacts its opposing effects on the trypanosome transcriptome. Nucleic Acids Res **43**:5501–5523.

12. **Ochsenreiter T**, **Cipriano M**, **Hajduk SL**. 2007. KISS: the kinetoplastid RNA editing sequence search tool. RNA **13**:1–4.

13. **König J**, **Zarnack K**, **Rot G**, **Curk T**, **Kayikci M**, **Zupan B**, **Turner DJ**, **Luscombe NM**, **Ule J**. 2010. iCLIP reveals the function of hnRNP particles in splicing at individual nucleotide resolution. Nat Struct Mol Biol **17**:909–915.
